# NADPH Oxidases (NOX): An Overview from Discovery, Molecular Mechanisms to Physiology and Pathology

**DOI:** 10.3390/antiox10060890

**Published:** 2021-06-01

**Authors:** Annelise Vermot, Isabelle Petit-Härtlein, Susan M. E. Smith, Franck Fieschi

**Affiliations:** 1 Univ. Grenoble Alpes, CNRS, CEA, Institut de Biologie Structurale, 38000 Grenoble, France; annelise.vermot@gmail.com (A.V.); isabelle.petit-hartlein@ibs.fr (I.P.-H.); 2 Department of Molecular and Cellular Biology, Kennesaw State University, Kennesaw, GA 30144, USA; ssmit325@kennesaw.edu

**Keywords:** reactive oxygen species, membrane protein, electron transfer, modular proteins, signaling molecule, oxidative stress

## Abstract

The reactive oxygen species (ROS)-producing enzyme NADPH oxidase (NOX) was first identified in the membrane of phagocytic cells. For many years, its only known role was in immune defense, where its ROS production leads to the destruction of pathogens by the immune cells. NOX from phagocytes catalyzes, via one-electron trans-membrane transfer to molecular oxygen, the production of the superoxide anion. Over the years, six human homologs of the catalytic subunit of the phagocyte NADPH oxidase were found: NOX1, NOX3, NOX4, NOX5, DUOX1, and DUOX2. Together with the NOX2/gp91^phox^ component present in the phagocyte NADPH oxidase assembly itself, the homologs are now referred to as the NOX family of NADPH oxidases. NOX are complex multidomain proteins with varying requirements for assembly with combinations of other proteins for activity. The recent structural insights acquired on both prokaryotic and eukaryotic NOX open new perspectives for the understanding of the molecular mechanisms inherent to NOX regulation and ROS production (superoxide or hydrogen peroxide). This new structural information will certainly inform new investigations of human disease. As specialized ROS producers, NOX enzymes participate in numerous crucial physiological processes, including host defense, the post-translational processing of proteins, cellular signaling, regulation of gene expression, and cell differentiation. These diversities of physiological context will be discussed in this review. We also discuss NOX misregulation, which can contribute to a wide range of severe pathologies, such as atherosclerosis, hypertension, diabetic nephropathy, lung fibrosis, cancer, or neurodegenerative diseases, giving this family of membrane proteins a strong therapeutic interest.

## 1. Introduction

The Nicotinamide Adenine Dinucleotide Phosphate (NADPH) Oxidases (NOX) family, considered a major source of ROS in eukaryotic cells, comprises seven members involved in various essential human physiological functions.

Although all NOX isoforms share structural homology based on a common catalytic core composed of six transmembrane helices chelating two hemes, as well as a dehydrogenase domain (DH) which binds the non-covalently linked flavin cofactor (FAD) and the NADPH substrate, they differ according to their cellular and tissue distribution, mechanism of activation or regulatory system.

**Figure 1:**
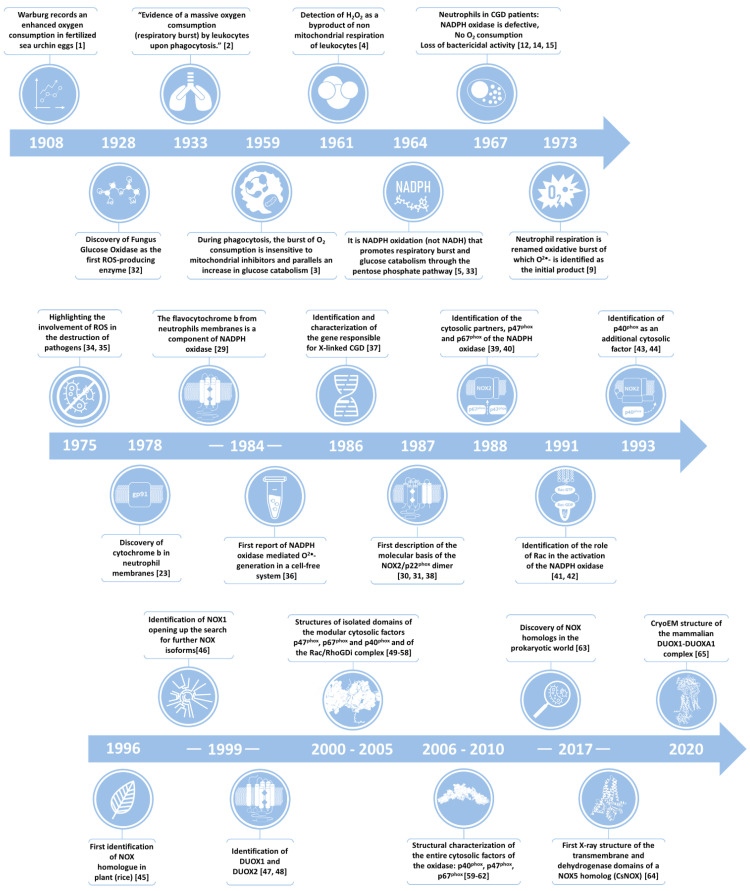
Timeline of the major steps leading to the identification and mechanistic description of the NADPH oxidase family of enzymes, specialized in the deliberate production of ROS [1,2,3,4,5,9,12,14,15,23,29,30,31,32,33,34,35,36,37,38,39,40,41,42,43,44,45,46,47,48,49,50,51,52,53,54,55,56,57,58,59,60,61,62,63,64,65].

Thus, this diversity allows these enzymes to be involved in numerous cell functions conferring a growing interest to the NOX family over the past decades (Figure 1).

## 2. NOX Family of NADPH Oxidases: Discovery of the Phagocytic Enzyme and History of NOX

In 1908, in a study on sea urchin fertilization, Warburg described an enormous oxygen consumption event (reasonably interpreted as respiration) at the fusion stage of the spermatocyte with the oocyte [1] This phenomenon was later attributed to the activity of the DUOX Udx1 homolog. The occurrence of a similar event during phagocytosis followed in 1932 [2] and was better characterized in 1959 [3].

This overconsumption of oxygen was initially attributed to increased mitochondrial activity to supply the energy required for the pathogen’s engulfment within the phagocytic vacuole [3].

However, classical inhibitors of mitochondrial respiration such as cyanide, azide or antimycin A did not inhibit this ‘respiratory burst’, observations which led to the identification of an unconventional alternative glucose-dependent respiration leading to the generation of H_2_O_2_ within the phagosomes of neutrophils (1961) [4].

A few years later, the existence of a NADPH oxidase [5], exhibiting a 100-fold selectivity of NADPH over NADH [6,7], was demonstrated and a myeloperoxidase (MPO) was shown to contribute to the ROS production resulting in antimicrobial activity [8]. This phenomenon was finally referred to as the oxidative burst.

Subsequent studies identified superoxide as the initial product resulting from NOX activity [6,9]. Upon natural dismutation or superoxide dismutase (SOD) activity [10], superoxide converts to H_2_O_2_, which constitutes the precursor of various bactericidal secondary ROS, such as HOCl generated by MPO. The identification and purification of the oxidase responsible for superoxide production in phagocytic cells, however, presented greater difficulties.

In parallel with molecular studies, clinical research on chronic granulomatous disease (CGD) [11], a rare immunodeficiency syndrome, also greatly contributed to the discovery of, and progress towards understanding, NOX [12]. The CGD phenotype usually appears in young children (with greater prevalence in boys), who suffer from recurrent infections; excessive accumulation of immune cells that cannot eliminate encapsulated pathogens leads to the formation of the eponymous granulomas [13]. Leukocytes of CGD patients perform chemotaxis, phagocytosis and degranulation, but the lack of superoxide production (and concomitant lack of oxidative burst) impairs their bactericidal function [12,14,15] (see Section 7.1 below for more details on CGD). Identification of the genetic lesion(s) responsible for CGD remained unknown for decades.

Initial suggestions for the enzyme responsible were eventually discarded [16]. Later, despite the successful production of an active NADPH oxidase solubilized from stimulated cells [17,18], purification of the protein presented problems because of its instability when removed from the membrane [19].

Although the presence of an unconventional type *b* flavocytochrome was observed in rabbit neutrophil granules in the early 1960s [20], this enzyme was initially attributed to an inactive form of cytochrome P_450_ and remained long unnoticed within the scientific community. This flavocytochrome was absent in leukocytes of CGD patients [21,22] and was insensitive to conventional inhibitors of mitochondria and myeloperoxidase [3,16].

Nonetheless, for many years the scientific community displayed a strong reluctance to adopt the hypothesis that this flavocytochrome was the NADPH oxidase.

**Figure 2:**
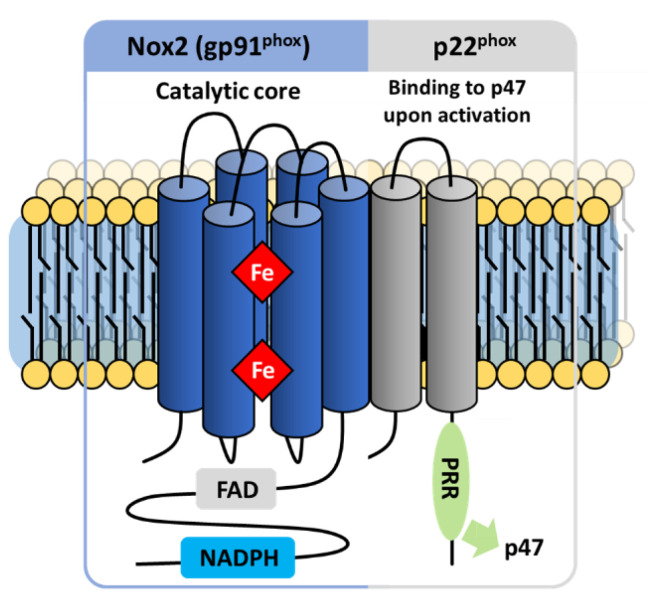
Catalytic subunit of the NADPH phagocyte oxidase complex. NOX2 topology harbors 6 membrane-spanning helices inter-connected by intra and extracellular loops, as well as a cytosolic domain enclosing FAD and NADPH-binding domains. Helices 3 and 5 of NOX2 chelate two b-type heme groups. Despite various studies, the number of transmembrane segments of p22^phox^ is not clearly identified, and the protein has thus been represented in this review with 2 helices and a C-ter cytosolic segment presenting a PRR domain that interacts with the cytosolic factor p47^phox^.

In 1978, Segal and Jones [23,24] identified this flavocytochrome *b* in neutrophil membranes and showed that it was deficient in CGD patients, thus explaining the impaired functions of CGD leukocytes. They showed that the protein contained two hemes and one FAD [25,26,27,28]. Initially referred to as flavocytochrome *b*_245_ based on its redox potential, the enzyme was later renamed as cytochrome *b*_558_ because of its characteristic absorbance at 558 nm in difference spectra, distinguishing this cytochrome from those of the endoplasmic reticulum and mitochondria [29]. The heterodimeric character of the membrane component of phagocytic NADPH oxidase (called phox) (Figure 2) was demonstrated by the identification of a 22 kDa protein (p22^phox^) that copurified with flavocytochrome *b*_558_ (Table 1) [30,31].

NADPH oxidase was also commonly referred to as gp91^phox^ because isolated from phagocytes it appears at an apparent molecular weight of 91 kDa on SDS PAGE gels. When the coding sequence for the enzyme was identified, Western blots allowed for confident assignment of the enzyme to the 91 kDa band [66]. Glycosylation of the enzyme explained the discrepancy in its apparent molecular weight from its predicted molecular weight of 58 kDa. Eventually, a new naming convention resulted in the official name of NOX2.

The development of cell-free systems [36], in which cytosolic fractions or lipids activated membrane fractions containing the flavocytochrome *b*_558_, provided both evidence for a larger NADPH oxidase protein complex and the necessary tools for the characterization of its component proteins [36,39,40,67]. Cell-free systems allowed for the discovery of the organizing cytosolic protein p47^phox^, the activating p67^phox^ [39,40] and also led to the demonstration of the roles of small GTP-binding proteins Rac1 (monocytes and macrophages) and Rac2 [41,42] (neutrophils) in activating the NOX2 complex [68]. Later on, the third cytosolic factor p40^phox^ was also identified [43]. Furthermore, investigators showed that lack of expression of any of the NOX complex components or the Rac activators leads to a CGD phenotype [69,70].

While the term NOX specifically refers to the transmembrane catalytic protein (gp91^phox^), it is sometimes used by extension to refer to the entire enzymatic multiprotein complex [71]. Thus, the phagocytic NADPH oxidase is commonly identified by the term NOX2.

Subsequently, the development of sensitive assays allowed for the detection, in non-phagocytic cells, of lower levels of ROS [72]. This breakthrough raised the suggestion that NOX-derived ROS production may occur in cell types other than neutrophils and that more diverse physiological processes could be involved, though the exact origin remained uncertain. Subsequent investigations revealed enzyme systems analogous to the phagocytic NADPH oxidase in a wide variety of cells such as fibroblasts [73], certain tumor cells [74] and vascular tissue cells [75]. Several teams concomitantly discovered NOX1 the first homolog of NOX2 (gp91^phox^), initially termed Mox1 (mitogenic oxidase 1) [46], NOH-1 (NADPH Oxidase Homolog-1) [76] and gp91-2 [77], confirming the hypothesis activity in non-phagocytic cell types. The number 1 of the NOX1 isoform was retained to facilitate its identification with the initial term of Mox1. In accordance with this new name, gp91^phox^, although it is the historical isoform, was renamed/termed NOX2.

The advent of genomics in the 2000s provided the means to search for sequences homologous to NOX2. Thus, the identification of NOX1 was quickly followed by the cloning of a new set of homologs: NOX3 [77], NOX4 [78] and NOX5 [79] as well as DUOX1 and DUOX2 (DUal OXidase) [47,48]. NOX5 and DUOX contain domains in addition to the catalytic core shared by all NOX isoforms [80].

Depending on the cell type, the various isoforms of NOX localize to a variety of membranes, including the plasma membrane and a variety of internal membranes such as the endoplasmic reticulum [81], nucleus [82], and mitochondria [83]. For decades, NADPH oxidases were considered to be exclusive to the eukaryotic kingdom, but the recent identification of prokaryotic homologs [63] has expanded our understanding that the enzyme appears in all divisions of life.

## 3. Components of the Phagocytic NADPH Oxidase Complex

### 3.1. NOX2: NADPH Oxidase Prototype

NOX2, expressed in phagocytic cells, was the first NOX isoform identified [84]. The other human isoforms and homologs in other organisms exhibit varying cellular localization, activation mechanisms, etc., but they all share a common catalytic subunit very similar to that of NOX2. The extensive biochemical characterization of the NOX2 enzyme constituted a fundamental prerequisite towards understanding the functional aspects of the whole family. Regardless of its place in biological evolution, because of its place in the order of discovery and its importance to human health, NOX2 serves as the prototype enzyme for the family. Although NOX2 is now understood to participate in various physiological processes such as signal transduction, angiogenesis or cell death [85,86,87,88,89], for many years the only known role for NOX2 was in innate immunity, so this function similarly provides the prototype for the family.

**Figure 3:**
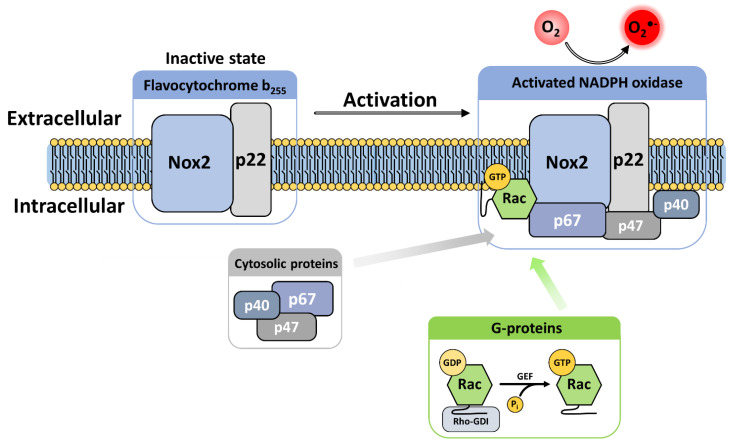
Activation process of the phagocytic NADPH oxidase. Detection of a pathogen triggers signaling pathways that lead to the phosphorylation of the cytosolic factors (mainly p47^phox^), inducing their translocation to the membrane-bound components of NOX2 and initiating the catalysis of superoxide production. Similarly, Rac-GDP sequestered in cytosol by RhoGDI is transferred to the membrane and its GDP exchanged for GTP for final assembly with p67^phox^, leading to NOX2 activation.

The phagocytic NADPH oxidase consists of a multicomponent complex involving the transmembrane flavocytochrome *b*_558_—which is the heterodimeric assembly of NOX2 and p22^phox^—supported by cytosolic protein factors p47^phox^, p67^phox^ and p40^phox^ and small GTP-binding proteins (G proteins Rac1 or Rac2) [90,91]. The diverse components are maintained physically dissociated in an inactive state in the absence of microbial infection. Upon activation, the regulatory subunits translocate to the membrane, where they assemble with the flavocytochrome *b*_558_ (Figure 3) [92]. These mechanisms provide tight regulation of NOX activity and avoid excessive production of superoxide and detrimental oxidation of biological macromolecules.

After activation and assembly, NOX2 catalyzes sequential vectorial electron transfer across the plasma membrane (which pinches off to form the phagosome), reducing O_2_ to synthesize the superoxide anion in the phagosome [89,93].

The initial superoxide production results in the formation of multiple secondary oxidative metabolites. Notably, superoxide dismutates to H_2_O_2_ which is typically further converted by MPO into HOCl, a potent bactericidal compound that is mainly responsible for pathogen clearance in immune cells [8].

### 3.2. The Components of the NADPH Oxidase Complex

#### 3.2.1. The Flavocytochrome *b*_558_

The flavocytochrome *b*_558_ defining the NOX catalytic core exists as a heterodimeric complex of the NOX2 and p22^phox^ proteins in a 1:1 stoichiometry (Figure 2). The study of neutrophils from patients suffering from CGD, with a loss of superoxide production, revealed that a deletion of either of these two subunits ultimately leads to the absence of the other within the membrane, indicating their mutually stabilizing interaction [94].

##### NOX2

**Figure 4:**
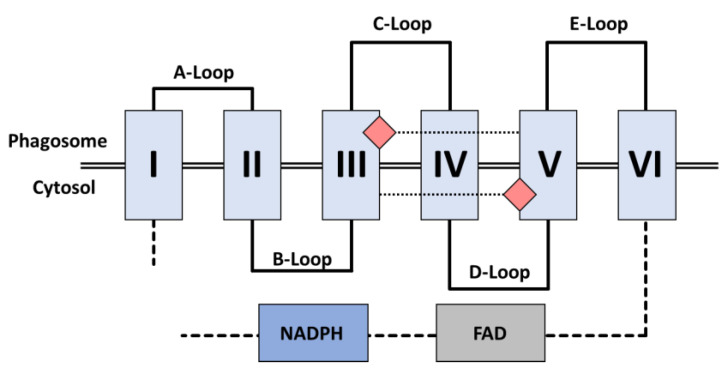
Organization of NOX2 transmembrane helices. Helices are numbered in the N to C direction. Two hemes are coordinated by conserved bis-histidyl motifs on helices III and V. (**B**) and (**D**) loops face the cytosol and contact the cytosolic DH domain. (**A**, **C**, **E**) loops face the extracytosolic space, which is equivalent to the interior of the phagosome, where oxygen reduction occurs.

NOX2 operates the transfer of electrons across the membrane, from cytosolic NADPH to molecular oxygen. The analysis of the NOX2 primary structure revealed the presence at the N-ter of a ferric reductase (FRD) transmembrane (TM) domain that encompasses six membrane-spanning helices connected by five inter-helix loops designated from A to E, from the amino-terminal extremity to the carboxyterminal end. The A, C and E loops face the extracytoplasmic side of the membrane (corresponding to the internal side of the phagosome). The B and D loops face the cytosol and are in close contact with elements of the C-ter dehydrogenase (DH) domain (Figure 4). Because of these contacts, the B and D loops likely participate in controls on NOX2 electron transfer, although their exact functions are not well established [95,96].

The transmembrane domain encompasses two strictly conserved pairs of canonical bis-histidyl heme binding motifs. The pairs reside on the 3rd and the 5th helices; on helix 3 the histidines are spaced 14 residues apart (His101 and H115), while on helix 5 they are 13 residues apart (His209 and H222). The imidazole rings of these residues provide the axial and distal ligands to the irons of two B-type hemes, holding them perpendicular to the plane of the membrane [97]. The proximal heme (closest to the cytoplasm) has a redox potential of −225mV, while the distal heme (furthest from the cytoplasm) has a redox potential of −265mV [98].

The cytosolic DH domain of NOX is homologous to the flavoenzyme ferredoxin-NADP^+^ reductase (FNR); like other FNR family members, it includes two subdomains: A β-barrel housing the FAD cofactor and a Rossman-fold that binds NADPH.

##### Electron Transfer

**Figure 5:**
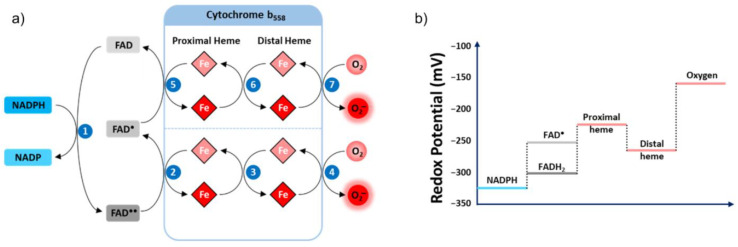
Mechanism of electron transfer catalyzed by the cytochrome *b558* of NADPH oxidase. (**a**) The NADPH substrate provides two electrons that are transferred to the FAD, reducing it to FADH2. The FADH2 transfers a first electron to the proximal heme of the cytochrome, which is rapidly transmitted to the distal heme and then to molecular oxygen, forming superoxide. The second electron carried by the FAD cofactor is transferred in the same way with the FAD as an initial donor. The order of successive steps is indicated in blue circles. (**b**) Redox potentials of the different couples that participate in NOX-catalyzed electron transfer.

NOX2’s transmembrane electron transfer can be decomposed into seven distinct steps (Figure 5a). This process is initiated with a hydride transfer originating from cytosolic NADPH, reducing the non-covalently bound FAD cofactor into FADH_2_ [99,100]. The second step constitutes the transfer of one electron from the reduced FAD (FADH_2,_ E°’ = −304 mV) to the proximal heme (E°’ = −225 mV) (Figure 5b) generating the semiquinone radical form of the FAD (FAD). The electron moves from the proximal heme to the distal heme during Step 3, then to molecular oxygen as the final acceptor (E°’ = −160 mV), resulting in the formation of superoxide anion (Step 4).

Once Step 3 is completed, the proximal heme is available to undergo Steps 5–7, recapitulating Steps 2–4. The difference is the donor initiating the electron transfer in Step 5 is now the semiquinone FAD (E°’ = −256 mV). The semiquinone possesses a diminished redox potential compared to FADH_2_, consequently supplying a lower driving force for the reduction of internal heme.

The respective redox potentials of the internal and external hemes unexpectedly make the transmission of the electron between the two heme groups energetically unfavorable [16,89,101,102]. While the functional significance of such an unforeseen architecture within the enzyme is not understood, this could argue the absolute necessity of oxygen as an acceptor to ensure a rapid flow of electrons through NOX, essential to preventing the accumulation of electrons in the proximal heme. Indeed, the reaction rates measured in anaerobic condition for both the flavin and the heme group were reported to be 1000-fold slower than in aerobic condition [103,104].

Study of the interaction between oxygen and the external heme suggests that the reduction of O_2_ occurs via an outer sphere mechanism [97,105,106] in which oxygen is not directly physically coordinated with the ferric ion of the distal heme but receives the electron by indirect saltatory transfer from the heme periphery. This model is consistent with the insensitivity of the oxidase to classical inhibitors of respiration such as carbon monoxide, azide or cyanide and confirms the absence of a free position on the heme iron.

##### The p22^phox^ Membrane Partner

Although the NOX2 protein exclusively performs electron transfer, p22^phox^ maintains stability [89,107]. The p22^phox^ scaffolding protein contains an affinity anchoring site for the cytosolic partner p47^phox^, and also indirectly for p67^phox^, p40^phox^ and the small GTPases Rac [95,108] through their binding with p47^phox^. Similar to Nox2, p22^phox^ is required and forms a heterodimer with NOX1,3, et 4 (Bedard and Krause, 2007).

Few direct structural data exist for p22^phox^. Biochemical characterization and subsequent partial crystal structures revealed a cytosolic C-ter domain enclosing a proline-rich region (PRR) that describes a PXXP pattern forming a polyproline helix II (PPII) [55,109,110], required for NOX activity through interaction with the p47^phox^ organizing subunit. Predictive algorithms and experimental evidence including monoclonal antibody epitope mapping [111], peptide walking [112] and analysis of expression in CGD patients [113] led to models with two or four membrane-spanning helices, putting both the N-ter and C-ter on the intracellular side of the membrane. Despite various studies, the number of transmembrane segments of p22^phox^ is not clearly identified, and the protein has thus been represented with 2 helices in this review. Phenotypic analysis associated with various mutations led to a 3-transmembrane structural model of p22^phox^. In this model, the C-ter is cytosolic, while the N-ter and the loop interconnecting helices 2 and 3 are extracellular [114]. So far, experimental evidence favors a two or four helical model of p22^phox^.

Specific deletions in p22^phox^ evidenced the joint involvement of its N-terminal 11 residues in both maturation and activity emphasizing the functional importance of this highly conserved region [115]. Conversely, deletion of the 54 C-ter residues leads to an impaired NOX2 catalytic activity but preserves NOX2 maturation.

**Figure 6:**
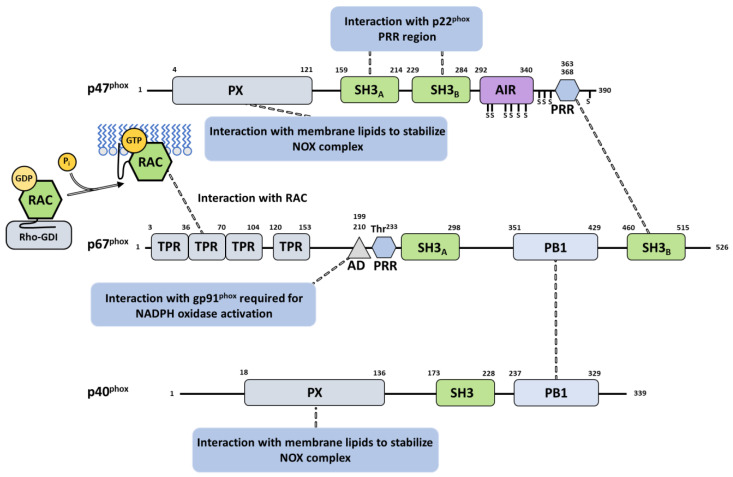
Diagram of the cytosolic factors of NADPH oxidase and their interactions with partners. The p47^phox^ and p40^phox^ proteins are initially self-inhibited and require phosphorylation to reach an active conformation. The p67^phox^ and p40^phox^ factors interact through their respective PB1 domains; the SH3B domain of p67^phox^ binds to the PRR domain of p47^phox^. After phosphorylation, the bis-SH3 domain of p47^phox^ is unmasked, triggering translocation of the p47^phox^–p67^phox^–p40^phox^ trimeric complex via interaction with the PRR domain of p22^phox^. At the membrane, the PX domains of p47^phox^ and p40^phox^ bind to membrane lipids. Rac-GDP sequestered in cytosol by RhoGDI is transferred to the membrane and GDP is exchanged for GTP, leading to the interaction with the TPR domain of p67^phox^. Domain boundaries are indi-cated by position numbers. Important phosphorylation sites in the AIR region of p47^phox^ are indicated as ‘S’; the major phosphorylation site of p67phox Thr233 is labeled. Dotted lines represent the inter-domain interactions. Adapted from [110].

The naturally occurring mutation P156G has no direct influence on the expression of p22^phox^ but prevents the translocation of cytosolic components to the membrane after neutrophil activation. This mutation highlighted the close involvement of three conserved residues in the interaction with p47^phox^: P152 and P156, which bind the N-ter SH3 motif of p47^phox^, and P158, which interacts with the C-ter SH3 domain of p47^phox^ [116] (Figure 6). Additional proline residues of p22^phox^ (P151, P155, P157, P160) also interact with p47^phox^, although they exert less influence [117]. These observations converge with the essential function of p22^phox^’s PRR in recruitment of p47^phox^ and subsequent assembly [115].

### 3.3. Cytosolic Components

#### 3.3.1. Cytosolic Factors

Each of the three phox proteins is a multidomain protein, and each undergoes complex rearrangements for interaction and function. p47^phox^ comprises an N-terminal PX domain that interacts with lipids; a bis-SH3 domain that binds p22^phox^; an autoinhibitory region (AIR); and a C-terminal proline-rich region (PRR) [89,110]. The activator p67^phox^ presents four successive tetratricopeptide repeat (TPR) motifs at the N-ter extremity, constituting the Rac-binding region; a highly conserved activation domain (AD); a first SH3 domain; a “Phox and Bem 1” (PB1) domain; and a C-terminal SH3 domain interacting with p47^phox^ [49,54,62]. p40^phox^, starting from the N-terminus, possesses a PX domain, an SH3 domain, and a PB1 domain [60] (Figure 6).

**Figure 7:**
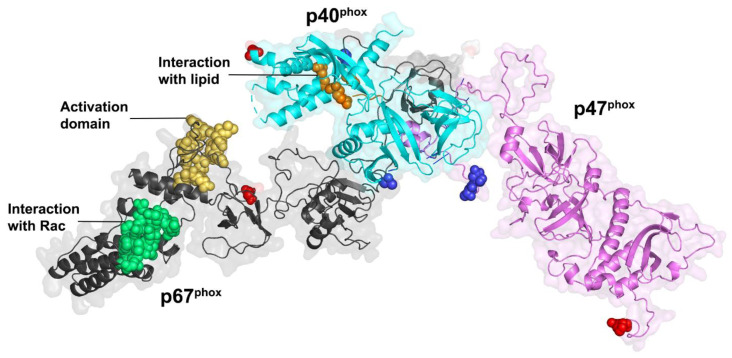
Model of the trimeric cytosolic complex of NADPH oxidase in the resting state [121]. Three-dimensional model of the heterotrimer: p40^phox^ (cyan ribbons), p47^phox^ (light purple ribbons), p67^phox^ (gray ribbons). The N-ter extremities are shown in red and the C-ter ends in blue. In p67^phox^, green spheres represent the β hairpin of the Rac interaction (residues within 115–130), yellow spheres the activation domain (residues within 199–210 [122]). The residues of the lipid interacting PX domains in p40^phox^ are represented by the orange spheres (R58, K92 and R105 [51]). Figure inspired by [121].

In unstimulated phagocytic cells, the cytosolic subunits p47^phox^, p67^phox^ and p40^phox^ associate in a soluble trimeric complex in which p67^phox^ associates to p40^phox^ via a PB1–PB1 interaction [118,119] and binds with p47^phox^ through an independent interaction of its SH3 C-ter domain with the PRR of p47^phox^ [108]. p47^phox^ is locked in a closed conformation thanks to several interactions between its various domains [59]. These interactions help prevent the cytosolic trimeric complex from interacting with the cytochrome b_558_ heterodimer at the membrane [120]. Similar to p47^phox^, p40^phox^ also exists in an autoinhibited conformation whose release could also contribute to membrane assembly [60]. Recently, the integrative characterization of the p47^phox^–p67^phox^–p40^phox^ complex [121] combining studies by FRET (Förster-type resonance energy transfer) imaging, FCSS (Fluorescence Cross Correlation Spectroscopy) and small angle X-ray scattering (SAXS), resulted in a structural model of the cytosolic trimer (Figure 7). The complex displays an elongated shape with a flexible region separating two domains ideally positioned for NOX activation and interaction with membrane components.

Cellular activation leads to phosphorylation of phox components, which induces conformational changes that release different interaction regions, leading to translocation to the plasma membrane and assembly with the cytochrome b_558_ heterodimer.

#### 3.3.2. G Proteins

Activation of the phagocyte NADPH oxidase complex also requires small G proteins, namely, Rac and Rap1A GTPases. Rac and Rap1A share a core structure that includes a N-terminal guanosine-binding site [123,124], a geranylgeranyl tail [125,126] and a C-terminal polybasic region that promotes anchoring of the GTPase to the membrane after prenylation [127,128]. Under the regulation of Guanosine Exchange Factor (GEF), the GTPase alternates between a GDP-bound inactive conformation and a GTP-bound active conformation.

In mammals, Rac exists in three isoforms, Rac1, Rac2 and Rac3 [89]. In monocytes and macrophages, all are expressed, but Rac2 predominates in human neutrophils [110].

**Figure 8:**
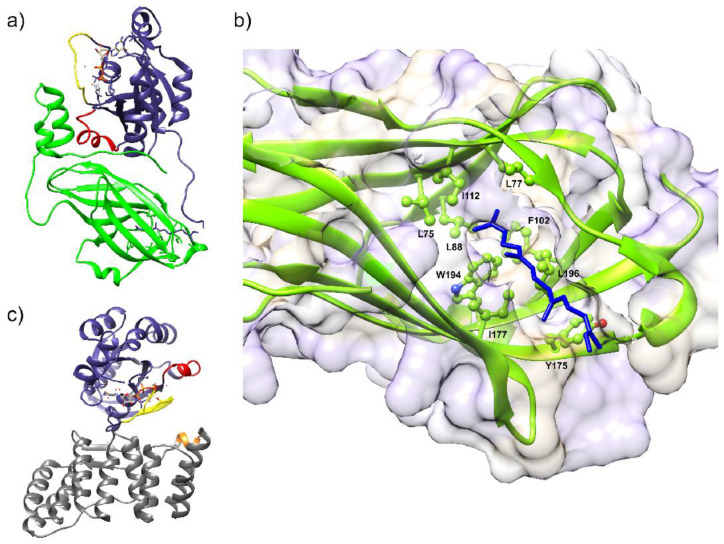
Atomic structure of Rac in interaction with RhoGDI or with p67^phox^. The structure was solved by X-ray diffraction at a 2.7Å resolution [50]. (**a**) Rac is represented in blue ribbon and RhoGDI in green. The GDP molecule is depicted in stick (white) within Rac, while the geranylgeranyl tail of Rac is represented as stick (dark blue) within RhoGDI. The switch I and II regions of Rac are highlighted in yellow and red, respec-tively. (**b**) Binding pocket of the geranylgeranyl tail. The Rac geranylgeranyl tail, represented in blue stick, binds in a hydrophobic cavity formed by RhoGDI, represented in green ribbon and surface. The hydrophilic regions of the RhoGDI-binding pocket are colored in purple, hydrophobic regions in orange. The hydrophobic residues of RhoGDI involved in interactions with the lipidic group are labeled. (**c**) Atomic structure of Rac in complex with p67^phox^. The structure of the Rac-p67^phox^ complex (1E96) [49], solved by X-ray diffraction at a 2.4Å resolution, was structurally aligned with the structure of the N-ter extremity of p67^phox^ (1HH8) [50]. Rac is represented in blue ribbon with switch I and II regions highlighted in yellow and red, respectively. p67^phox^ is represented in gray ribbon, the activation domain is represented in orange.

In the absence of activation signal, Rac is sequestered into the cytosol by association with the RhoGDI inhibitor protein (Rho GDP-dissociation inhibitor), which masks the geranylgeranyl tail of Rac within a hydrophobic pocket (Figure 8) [50]. After the cell receives an activation signal, phosphorylations lead to Rac release from RhoGDI, its geranylgeranyl tail insertion within the plasma membrane, and to interaction with p67^phox^ (Figure 8c) and NOX2 in the NOX2 complex assembly [49].

Rap1A, discovered by co-purification with cytochrome *b_558_*, is a small G protein abundant in neutrophils and colocalized and translocated with flavocytochrome *b* in resting and activated neutrophils [50,129,130]. The GTPase also harbors a phosphorylation site leading to the inhibition of the association of Rap1A with flavocytochrome *b*_558_ upon stimulation by protein kinase A (PKA) [131]. The role exerted by Rap1A in NOX activity has not been yet fully identified; however, the loss of oxidase activity described after the immunodepletion of Rap1A in cell-free assays suggests a positive regulatory role of this subunit [132]. Neutrophils from Rap1A^−/−^ mice exhibited slowed, attenuated superoxide production after fMLF stimulation compared to neutrophils from WT, supporting the idea that Rap1A upregulates NOX activity [133].

## 4. The Activation Mechanisms and Assembly of Phagocytic NADPH Oxidase

### 4.1. Activation

Phagocytic NOX activation is one consequence resulting from several upstream cellular-level events. First, tissue-resident macrophages detect pathogenic micro-organisms and produce activating pro-inflammatory chemoattractant mediators at the infection site [134]. In response to these signals, neutrophils extravasate from the vasculature to migrate toward the epicenter of inflammation guided by the chemoattractant gradient.

Antigen recognition at the leucocyte membrane may occur via a direct interaction of micro-organism-specific motifs, highly conserved in the course of evolution, with a variety of membrane receptors such as Toll-like Receptor (TLR), C-type Lectin Receptor (CLR), Macrophage Mannose Receptor (MMR) and scavenger receptors [135]. Alternatively, recognition can be mediated through opsins, intermediary adaptor biomolecules that coat the surface of pathogenic cells and which enhance recognition [136].

Upon recognition, the phagocyte begins to engulf the pathogen and a signaling cascade is initiated to trigger the activation of NOX2 through the membrane recruitment of the multiple cytosolic partners of the NOX2 complex. This process ultimately ends with the release of diverse bactericide effectors (such as anti-bacterial peptides, proteases or ROS) into the phagosome where they kill the pathogen.

NOX2 activation requires the assembly of the multiprotein complex through a complex series of inter-protein, protein-lipid and intra-protein interactions. In vivo, activation requires phosphorylation to induce a conformational change to unmask p47^phox^’s autoinhibited tandem SH3 domain. Release of the tandem SH3 enables p47^phox^ translocation and binding to the PRR region of p22^phox^. This interaction provides a scaffold for p67phox and p40^phox^ assembly in the complex. Conformational changes induced by the phosphorylation of p47^phox^ and p40^phox^ also result in exposure of their respective PX domains, thus enabling the anchoring of the p47^phox^–p67^phox^–p40^phox^ trimeric complex to membrane phosphoinositides. When joined by Rac, this complex enables electron transfer and superoxide production in the presence of NADPH [110].

In reconstituted systems in vitro, excess of p67^phox^ subunit, associated to Rac (Figure 8c), was both necessary and sufficient for NOX2 catalytic activity [137]. In such systems, p47^phox^ was not essential for activation [138,139]. Thus, p47^phox^ appears mainly to exert an organizing role, recruiting the different cytosolic partners to the membrane.

Studies in knockout mice showed that Rac2 was required for optimal activation of the respiratory burst in neutrophils [140,141], implying direct activation of the complex, although Rac1 and Rac2 can activate Nox2 to a similar extent in a cell-free system [41,42].

These detailed biochemical and structural characterizations of the cytosolic factors and Rac protein interactions and dynamics allowed for the development of engineered proteins that mimic the assembly of the NOX complex using a single chimeric fusion made of specific domains from p47^phox^, p67^phox^ and Rac [142,143,144,145]. Such chimeras have been used to further characterize assembly and activation of the NOX using cell-free systems and are now used as a tool for physiological studies in living cells to simply promote the NOX assembly and activity [146].

### 4.2. NOX Priming

Before NOX activation, multiple external activators, such as PAF^5^ (Platelet-Activating Factor) [147], fMLF (formyl-methionyl-leucyl-phenylalanine) [148], LPS (Lipopolysaccharide) and TNFα (Tumor Necrosis Factor) [149], or alternative particulate stimuli such as opsonized bacteria, cause the ‘priming’ of the enzyme. In vitro, these physiological inflammatory agents may induce either a priming effect at low concentrations (<10^−7^ M), or trigger direct neutrophil activation and the production of ROS at higher concentrations. This pre-activation does not elicit the respiratory burst but allows for an additional secondary stimulus to result in superior microbial killing. Priming leads to a typically faster and enhanced response, ensuring efficient clearance of pathogens during phagocytosis. This priming has been correlated to the phosphorylation of Ser 345 of p47^phox^. This allows for the action of Pin1 prolyl-isomerase on p47^phox^, thereby inducing conformational changes and facilitating additional phosphorylation by protein kinase C (PKC) in the activation process [150,151].

However, this process is likely to result in subsequent oxidative damages to surroundings tissues, ultimately promoting an uncontrolled inflammation when the intensity of the downstream response is not properly adapted [88].

### 4.3. Phosphorylation of Subunits of the NADPH Oxidase Complex

NOX2 activity is governed by a series of phosphorylations performed by several kinases, including PKC and mitogen activated protein kinase (MAPK), themselves activated by specific signaling pathways after pathogen capture and internalization.

**Figure 9:**
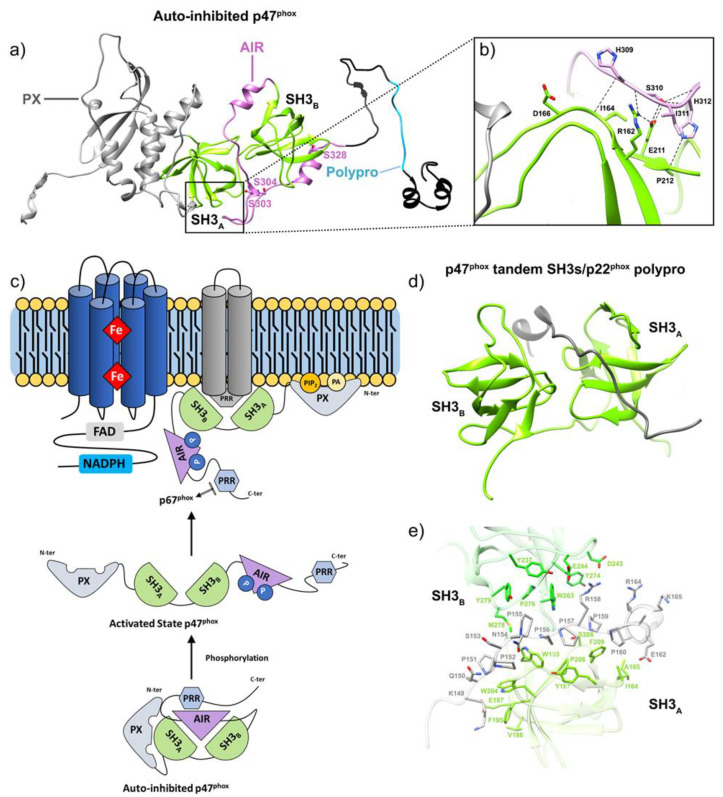
Model of the p47^phox^ activation mechanism showing the link between AIR and PX motif releases. (**a**) Model of the auto-inhibited p47^phox^. This model results from the combination of SAXS and HDXMS characterization on whole p47^phox^ [59,61] and the insertion of the high-resolution structure of the individual domains: the PX domain (pdb: 1KQ6), autoinhibited tandem SH3, comprising the two SH3 domains locked by the AIR region (pdb: 1NG2) [55] and the polyPro of p47^phox^ from pdb:1K4U [54]. The PX domain is in gray, tandem SH3 in green, AIR sequence in pink and C-terminus in black, except for the polyPro motif in blue. The phosphorylation target sites involved in the activation of p47^phox^ (S303/S304/S328) are represented in ball and stick. (**b**) The insert provides a close-up showing the network of interactions maintaining PX/SH3 and AIR locked alto-gether. Polar interactions occur between residues Arg-162, Ile-164, Glu-211, and Pro-212 from SH3A (green) and residues His-309, Ser-310, Ile-311, and His-312 from AIR (pink). The docking site of the PX domain in the resting state as shown by its release upon mutation on residue 162 and 166 [61]. (**c**) Phosphorylation of the AIR domain leads to the release of the auto-inhibitory intramolecular interaction between the AIR domain and the bis-SH3 domain, leading to the release of the PX domain and the activation of p47^phox^ [61]. (**d**) Structure of p22^phox^-p47^phox^ complex (pdb: 1WLP) solved by NMR [109]. The p47^phox^ tandem SH3s (aa 151–286) is represented in green ribbon and the p22^phox^ polyPro (aa 149–168) is represented in gray ribbon. This structure obtained with a truncated recombinant p47^phox^ bis-SH3 and p22^phox^ polyPro (residue 146–179) mimics the interaction between p47^phox^ and p22^phox^ following AIR release. (**e**) Recognition of p22^phox^-(149–168) (in gray) by the SH3A and SH3B (in green) domains of p47^phox^-(151–286). SH3A and SH3B (in green). The side chains of the amino acids involved in the recognition of p22^phox^-(149–168) by the SH3A and SH3B domains are shown in the wire model and labelled.

Upon pathogen recognition, kinases phosphorylate serines 303, 304 and 328 within the AIR of p47^phox^ [152,153]; these phosphorylations lead to the release of autoinhibition (Figure 9), the exposure of the PX and bis-SH3 domains and binding to lipids and p22^phox^, respectively [61,154]. Experiments using specific inhibitors concluded that the priming of neutrophil NADPH oxidase at inflammatory sites was mediated by the phosphorylation of p67^phox^ on Thr233 of its PRR region by the MAPKs [155]. PKC targets position Ser315 of p47^phox^ [152,156]; Thr154 phosphorylation on p40^phox^ also appears crucial for oxidase activation [157]. Finally, in vitro studies have shown that phosphorylation of p47^phox^ reinforces the binding of p67^phox^ to cytochrome b558 [90].

Similarly, after cellular activation, phosphorylation of RhoGDI on Ser101 and Ser174 causes Rac release [158]. Following release, a guanine nucleotide exchange factor (GEF) facilitates GDP/GTP exchange which in turn leads to exposure of the geranylgeranyl tail [159], thus triggering the recruitment of Rac to the membrane [129], independently of other cytosolic factors. Rac interacts with the p67^phox^ N-ter TPR (Figure 8c). Rac directly interacts with NOX2 via specific motifs, located in the region between residues 124 and 135 of Rac [160].

Multiple phosphorylations also modify the transmembrane components p22^phox^ and NOX2. Phosphorylation of p22^phox^ leads to phospholipase D production of phosphatidic acid, which participates in the elicitation of NOX2 activity [161,162,163]. Studies employing a specific PKC inhibitor (GF109203X) showed that PKC phosphorylates the DH domain of NOX2, leading to enhanced binding of the DH domain to Rac2, p67^phox^ and p47^phox^, and a faster electron transfer [152].

Recent work has unveiled that ATM kinase (Ataxia-Telangiectasia Mutated) phosphorylates Ser486 of NOX2, within the NOX Insertion Sequence region of its DH domain. Inhibition of ATM kinase led to an increase in the catalytic activity of NOX [164]. Similarly, the phosphorylation of NOX2 by PKA was demonstrated to negatively regulate superoxide production [165]. Hence, the PKC-mediated phosphorylation of NOX2 promotes the assembly and catalytic activity while ATM and PKA kinase-mediated-phosphorylation inhibits activity. These antagonistic effects testify to phosphorylation exerting a fine-scale regulation of NOX activity and superoxide production.

Stimulation of neutrophil membrane receptors activates the PI3-Kinase (PI-3K) that transfers an inorganic phosphate from ATP to the inositol of membrane phosphatidylinositols (PIs) to further catalyze the formation of essential PIs. Besides this function, PIP2 and PIP3 are also involved in the regulation of the PKC activity.

### 4.4. Activation by Lipids and Arachidonic Acid

PI3-K action initiates the formation of essential PI derivatives PI(3)P, PIP2 and PIP3 that bind the PX domain of p47^phox^ or p40^phox^ with high specificity [166] (Figure 9). Besides this function, PIP2 and PIP3 also participate in the regulation of PKC activity [167]. Ca^2+^ ions released downstream of IP3 binding to endoplasmic reticulum receptors allows for the activation of cytosolic Phospholipase A2 (PLA2) [168], an enzyme that catalyzes the production of arachidonic acid (AA). AA then participates in inducing several signaling molecules, such as PKC, involved in the subsequent elicitation of NOX [169]. AA is also suspected to induce a transient conformation of cytosolic factors that enables their association with flavocytochrome *b*, promoting optimal superoxide production [170,171].

Moreover, the direct action of AA can release the p47^phox^ bis-SH3 tandem domain from the AIR, thus promoting p47^phox^ interaction with p22^phox^ [172,173,174]. Exogeneous supply of AA in Rac2 knock-out neutrophils leads to activation of NOX, suggesting the lipid can in part replace the action of Rac on p67^phox^ [175]. Finally, anionic membrane phospholipids have been shown to be essential for NOX2 activation thanks to the use of chimeric cytosolic factors (see above) in the cell-free assay [176].

## 5. NOX Homologs and Isoforms

Initially considered a unique enzyme of vertebrate leucocytes operating in innate immunity [177], starting in 1999, investigators began to find other NOX isoforms in humans [89] and homologs in other organisms, including invertebrates and plants [45,178,179,180]. Here, we focus on human isoforms. Professional ROS production by NAPDH oxidases constitutes a process essential to many physiological mechanisms such as the regulation of vascular pressure, balance, cell growth, apoptosis, fertilization and angiogenesis through the existence of various NOX isoforms appearing heterogeneously in a wide variety of cells type and tissues. These isoforms perform distinct function through differential expression and regulation.

### 5.1. NOX1-NOX3

**Figure 10:**
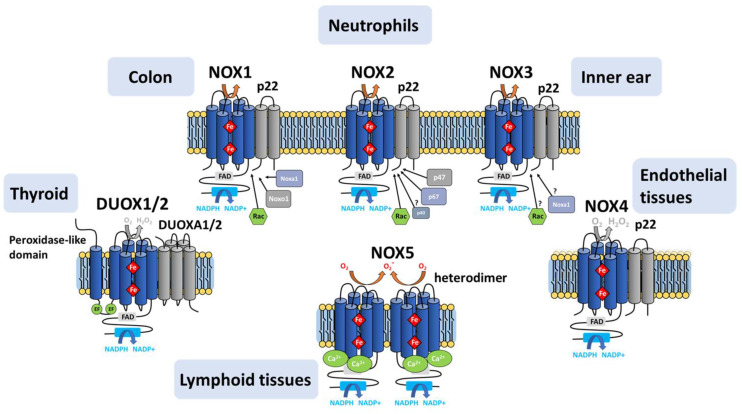
Representation of the NADPH oxidase isoforms. Despite their similar structure and enzymatic functions, the activation mechanisms of the NOX family enzymes differ. NOX1 activity requires p22^phox^, NOXO1, NOXA1 and the small GTPase Rac; NOX2 requires p22^phox^, p47^phox^, p67^phox^ and Rac. NOX3 requires p22^phox^ and NOXO1 and may require NOXA1 depending on the species; Rac can participate but its necessity for activity is not clearly established. NOX4 requires p22^phox^ in vivo and is constitutively active. NOX5, DUOX1 and DUOX2 are activated by Ca^2+^ ions; DUOX1 and DUOX2 require an association with the maturation factors DUOXA1/DUOXA2 for activation. NOX 1, 2, 3 and 5 produce mainly superoxide, Nox4 produces mainly H_2_O_2_, and DUOX1 and DUOX2 produce both [89].

NOX1 and NOX3 constitute the closest isoforms to phagocytic NADPH oxidase (Figure 10), sharing 60% sequence identity with NOX2. NOX1 constitutes the predominant isoform in the colon, prostate, uterus and vascular cells [46,76,181]. NOX3 is typically expressed in the inner ear. NOX3 expressed in cochlea produces ROS that has been linked to hearing loss [182], while NOX3 expressed in the vestibule produces ROS involved in gravity perception [181]. Low abundance of NOX3 has also been identified in the brain and lungs, albeit the function in these tissues is still elusive [183,184,185]. Like NOX2, NOX1 and NOX3 are both glycosylated in vivo [186,187].

Under physiological conditions, activation of NOX1 and NOX3 requires the presence of the cytosolic factors NOXO1 and NOXA1, respectively, homologous to the p47^phox^ and p67^phox^ NOX2 subunits [46,188].

However, in vitro studies revealed that p47^phox^ and p67^phox^ can substitute effectively for NOXO1 and NOXA1 in the assembly and activation of NOX1 [188] and NOX3 [189], suggesting a lack of absolute functional specificity in the interaction between NOX isoforms and their assembly partners [46,188,189].

### 5.2. NOX4

NOX4, which is highly expressed in the kidney, osteoclasts, fibroblasts and endothelial cells, shares a common catalytic core with NOX1 to 3, but shares only 39% identity with NOX2. Like NOX2, NOX4 maturation strictly depends on p22^phox^, as evidenced by studies in embryonic kidney cells knocked out for p22^phox^ expression [190,191]. NOX4 contains predicted glycosylation sites and some evidence exists for in vivo glycosylation [190,191,192,193].

Unlike other isoforms, NOX4 is constitutively active. Although this constitutive activity is presumably regulated by the cellular localization of NOX4, the involvement of activating factors such as protein disulfide isomerase (PDI), or the Poldip2 (factor) (Polymerase-δ Interacting Protein 2) was suggested [194,195,196] after these factors were found colocalized with the NOX4/p22^phox^ complex.

NOX4 produces H_2_O_2_ as the sole or vast majority of detectable ROS product even in vitro in the absence of superoxide dismutase [197]. The point mutation of His222 in the extracytoplasmic E-loop of NOX4 inhibits H_2_O_2_ production, implicating this residue in determining NOX4’s ROS species [198].

### 5.3. NOX5

NOX5 also shares a common core architecture with the other isoforms (27% identity with NOX2), with the addition of a N-ter extension containing 4 EF-hand motifs. NOX5 is endowed with other specificities compared to other NOX isoforms, such as a Ca^2+^-dependent activation, no requirement for p22^phox^ and cytosolic factors, and the absence of glycosylation [199,200].

Binding of calcium ions to NOX5’s extra EF-hand domain results in a conformational change of that domain which exposes hydrophobic regions that bind to the catalytic core to activate electron transfer [201]. NOX5 has relatively low sensitivity to Ca^2+^ on its own [202], but the binding of calmodulin at the C-terminal region of NOX5 DH triggers Ca^2+^-dependent conformation change [203] that augments its Ca^2^^+^ sensitivity, thereby enhancing ROS production in low Ca^2^^+^ concentrations. Similar to other isoforms of NOX, NOX5 is regulated by various post-translational modifications such as phosphorylations and oxidations. Finally, co-precipitation assays have shown the ability of NOX5 to form a functional homodimer through interactions between the two dehydrogenase domains [199].

### 5.4. DUOX1/DUOX2

The observation of NADPH and Ca^2+^-dependent hydrogen peroxide production in thyroid cells led to the discovery in 1999 of dual oxidases DUOX1 and 2 [47]. In addition to the NOX catalytic core like NOX1-4, and an EF-hand domain like NOX5 but with two instead of four EF motifs, DUOXes also contain an N-terminal extracellular peroxidase-like domain connected to the rest of the protein by an extra TM helix. In mammalian DUOX, absence of histidines implicated in heme chelation correlate with a lack of intrinsic peroxidase activity; *C. elegans* Duox does bind heme and shows a low level of peroxidase activity [204]. The DUOX glycosylation state correlates with its maturation and its ROS product [205,206,207,208].

DUOXes are sequestered in an inactive state in the endoplasmic reticulum. They require a maturation factor (DUOXA1 or DUOXA2) to adopt a conformation consistent with the acquisition of post-translational modifications responsible for the migration of the complex from the endoplasmic reticulum to the plasma membrane. It has been reported that in the presence of DUOXA2, the DUOX1 enzyme produces O_2_^●-^ while DUOX2 also produces H_2_O_2_ [209].

Despite the evidence in favor of a prokaryotic origin of NOX as early as 2004 in the independent studies of [210] and [211], the existence of prokaryotic NOX has only been recently confirmed.

## 6. NOX: From Bioinformatics to Structural Biology

### 6.1. Phylogenetic Analysis of Eukaryotic NOX

Canine leukocytes were the source for the first report of mammalian respiratory burst in mammals [2]; leukocytes from several other mammals were used for decades to investigate NOX biochemistry, indicating the early understanding of NOX enzyme homologs in mammals. The advent of large-scale sequencing of genomes provided the data that allowed NOX homologs to be identified with confidence in many other organisms, even those without leukocytes. The presence of bona fide NOX homologs in fungi, slime molds, red algae, and plants [212,213,214,215] indicated that NOX arose before the plant/animal split in evolution. Some animals appear to have orthologs of particular human isoforms, while other animals have NOX homologs that do not appear to fit neatly into the human (mammalian) isoform categories [96]. Physiological roles for NOX homologs in many species indicate a common function in stress responses [216].

**Figure 11:**
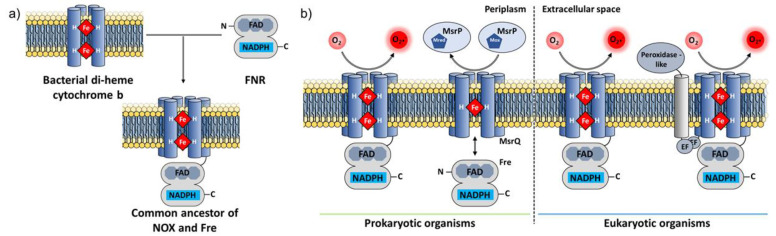
Emergence of proteins of the NOX or Fre family by the fusion of two ancestral genes. (**a**) The transmembrane domain homologous to cytochrome b is shown in yellow, and the cytosolic domain homologous to FNR in gray. Figure adapted from [110]. (**b**) Topological comparison between the two-component system MsrQ/Fre, the prokaryotic homologue SpNOX and eukaryotic NOX and DUOX. The FRD domain embedded in the membrane is shown in blue. The soluble FNR domains bearing the NADPH- and FAD-binding sites are in gray; EF hands and peroxidase-like domains are also shown. The electron acceptors and products of each system are also represented in the corresponding periplasmic/extracellular compartments. Adapted from [221].

The homology of the NOX DH domain to the FNR family [217] and of the NOX TM domain to mitochondrial cytochrome b and chloroplast b6f [218] were noticed early. The transient formation of a two-component system between FNR and cytochrome b6f demonstrated that a modular intermediate emerged during evolution [219,220]. Two unrelated, independent investigations reported the homology of the prokaryotic transmembrane protein YedZ (which lacks a DH domain) with the transmembrane domain of NOX and with the transmembrane domain of the STEAP family, which has an N-terminal DH-type domain and a C-terminal domain TM domain [80,210,211]. This TM domain common to all these transmembrane electron transfer enzymes has been renamed the Ferric Reductase Domain (FRD) [80,210,211]. The observations of homology naturally led to the hypothesis that an ancestral gene encoding a transmembrane b type cytochrome protein fused during evolution with a gene encoding an FNR protein, leading to the emergence of the common ancestor of the NOX family [110] (Figure 11a). Comprehensive searches failed at first to find prokaryotic NOX homologs [96,110]. In 2013, a bioinformatics analysis suggested the existence of NOX-like homologs in bacteria [80] but left the question of NOX function in prokaryotes open.

### 6.2. Experimental Discovery of Prokaryotic NOX

The functional significance of the YedYZ oxidoreductase complex—renamed MsrPQ once its methionine sulfoxide reductase function was discovered—was reported in 2015 [222] and more fully elucidated in 2017 [221]. In this system, the cytosolic flavin reductase protein Fre (FNR superfamily) that binds flavin and NADPH interacts with, and reduces the hemes of, the membrane-bound MsrQ protein (FRD superfamily) that ultimately reduces the MsrP molybdo-enzyme in the periplasm (Figure 11b). This direct observation of prokaryotic homologs of the separate NOX domains in action prompted a different, modular bioinformatic search approach using specific conserved NOX motifs. This search yielded not only approximately 1000 new NOX sequences from bacteria [63] but also the first demonstration of a bona fide functional NOX homolog in prokaryotes. The *Streptococcus pneumoniae* NOX (SpNOX) displays all the biochemical hallmarks shown by eukaryotic NOX but with the added benefit of robust activity in detergent solution.

### 6.3. Structural Characterization of NOX Proteins

**Figure 12:**
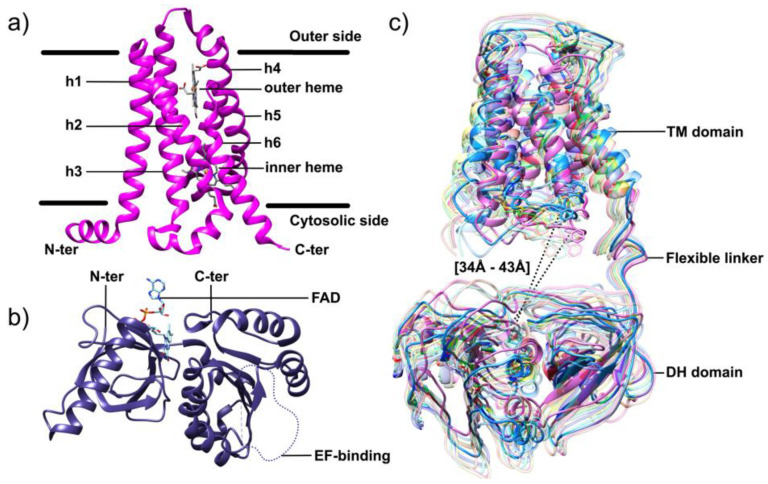
Structure of the DH and TM domains of CsNOX and the SANS structure of SpNOX. (**a**) TM domain of CsNox solved by X-ray crystallography at 2.05Å resolution [64]. The 6 transmembrane helices and the two chelated hemes are labelled. The positions of the bilipidic layer are indicated by the horizontal black lines. (**b**) The DH domain of CsNox was solved by X-ray crystallography at 2.2Å resolution. The FAD cofactor co-crystallized with the protein is la-belled and the unstructured EF-hand-binding loop is depicted in dotted gray (D611-T634). (**c**) Some of the conformations generated by Pepsi-SANS along Non-linear Normal Mode Analysis for SpNOX in a semi-transparent ribbon style [226]. The two most distant conformations have been represented in opaque ribbon; between these two configurations, the gap between the D-loop of the TM domain and the FAD-binding site varies from 34 to 43 A, highlighting the flexibility of the inter-domain linker.

Homology models of NOX DH domains based on similarity to multiple crystallized FNR family proteins [164,223,224,225] yielded a considerable amount of usable information for biochemical investigations. TM models of NOX, however, suffered from a dearth of similarity to crystallized proteins. Thus, the first high-resolution structural characterization of an algal NOX, CsNOX, was a milestone [64,97]. Crystallization of full-length CsNOX (an NOX5 homolog from the cyanobacterium *Cylindrospermum stagnale*) proved impossible, but the investigators instead produced and crystallized the DH (Figure 12a) and TM (Figure 12b) fragments independently. This first structure of an NOX TM domain provided support from a structural standpoint for the outer sphere mechanism of electron transfer from external heme to O2 [64]. These structures, and the in silico docked structure, provided new information, particularly a template for TM domain modeling, that could be transposed to eukaryotic NOX.

A combined SANS and molecular modeling study of SpNOX provided a first low-resolution structural characterization of a full-length NOX enzyme [226]. This investigation revealed a distinctly less compact structure than the docking of the CsNOX domains implied, and the SpNOX SANS data strongly suggested a flexible linker between the TM and DH domains (Figure 12c) as well as the potential for substrate and cofactor binding to contribute to an active conformation [226].

**Figure 13:**
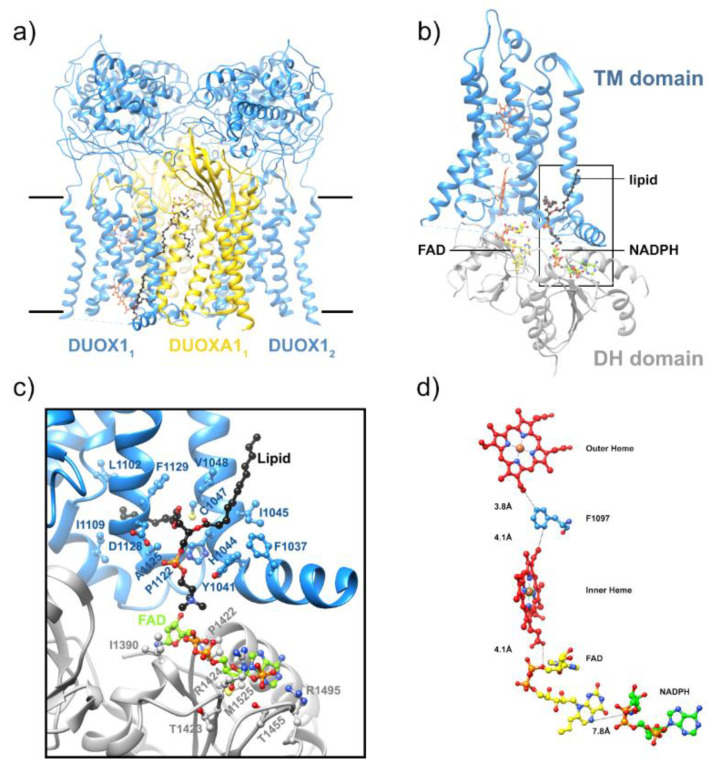
Structure of the mouse DUOX1/DUOXA1 complex solved by cryoEM in the inactive dimer of dimer configuration (3.2 Å) and in an active heterodimer state with NADPH at, respectively, (3.3 Å). (**a**) The structure of DUOX1 is displayed in blue and the structure of DUOXA1 is displayed in yellow. In the dimer of dimer configuration, the cryo-EM map allowed modeling of extracellular domains and TM domains, whilst cytoplasmic domains were too flexible to be resolved. (**b**) The TM domain of the active heterodimer state is displayed in blue and the DH domain is displayed in gray. The FAD, NADPH and nearby lipid molecules are shown as sticks and balls and colored in yellow, green and black, respectively. (**c**) The insert corresponds to the frame in black present in b) and provides a close-up view showing the interactions in the region of the lipid-mediated NADPH-binding site. (**d**) The electron transfer path deduced from the structure of the activated DUOX with calculated distances between players in the electron transfer path. Adapted from [65].

Most recently, the first high-resolution structures of a full-length enzyme of the NOX family, the mouse DUOX1/DUOXA1 complex, were solved by cryoEM [65], constituting a major leap forward. This study obtained two structures of mouse DUOX1, one in complex with DUOXA1 and one in an inactive dimer of dimers configuration. Similar to SpNOX [226], the inactive dimer-of-dimers state shows flexibility in the positioning of the cytosolic DH domain of DUOX1 (Figure 13a). This flexibility would seem to arise from the linker between the TM and DH domains, but this presents a paradox. The absolute length of the linker between the TM and DH domains is nearly constant in all mammalian NOX family members, including NOX4 homologs which are constitutively active. On the other hand, mammalian NOX4 homologs and the constitutively active bacterial SpNOX contain a proline at the N-terminal end of the predicted first beta strand of the DH domain, while other mammalian NOX are missing this proline. The proline may alter the DH domain to promote better docking to the TM domain. In any case, evidence continues to mount that the binding of other molecules, including substrate and cytosolic factors, leads to the repositioning of NOX domains for electron flow.

As noticed in the CsNOX structure [64], a lipid molecule in the DH domain confirms the idea of a lipid-mediated NADPH-binding pocket (Figure 13b). This agrees with many reports regarding the role of specific lipids and amphiphiles in the activation of NOXes [36,227,228,229,230,231]. These observations suggest that the additional proteins, or domains, that some NOX isoforms require for activity could drive and stabilize the correct positioning of the DH domain onto the TM domain for efficient electron transfer. In addition, the cryo-EM structures revealed a ~6° tilt of the TM domain during the transition to an activated state that could potentially expose the O_2_ entering/H_2_O_2_ exiting path to the extracellular solvent.

Together, the recent structural studies represent a crucial advance in the understanding of NOX activation, regulation and ROS production. Because of NOX participation in myriad physiological and pathophysiological processes, the new structural knowledge sets the stage for translation to new and better drug discovery programs with NOX as targets.

## 7. Involvement of NOX in Physiological Processes

ROS are involved in many physiological functions, such as immune host defense and multiple cell signaling pathways. As enzymes specialized in the deliberate production of ROS, members of the NOX family directly contribute to such processes. In this section, the wide range of physiological implications directly modulated by the activity of NOX will be detailed to illustrate the pleiotropic role of these enzymes.

### 7.1. Involvement in Host Defense and Inflammation

Immune defense is a major function mediated by NOX, as evidenced by initial studies on chronic granulomatous disease (CGD) [38]. However, while superoxide production by NOX was initially presumed to be the unique underlying process responsible for bacterial destruction, it is now established that a successful ROS-mediated elimination actually results from an intricate cooperation between several mechanisms [72,232]. In addition, studies have underlined the existence of alternative ROS-independent killing processes supported by NOX enzymes [89,93,233].

#### 7.1.1. Phagocytosis: An ROS-Dependent Pathogen Clearance Process

Considerable efforts over the past decades to decipher the underlying mechanisms involved in immune defense during phagocytosis, and more specifically regarding the oxidative burst, has brought major advances towards the comprehension of NOX2 and the overall NOX family.

Upon bacterial infection, chemotactic compounds such as bacterial-derived fMLF and human-derived activated complement component (C5a) or interleukin 8 [234] tightly regulate many cellular activities, including the activation of the NADPH-oxidase [235].

**Figure 14:**
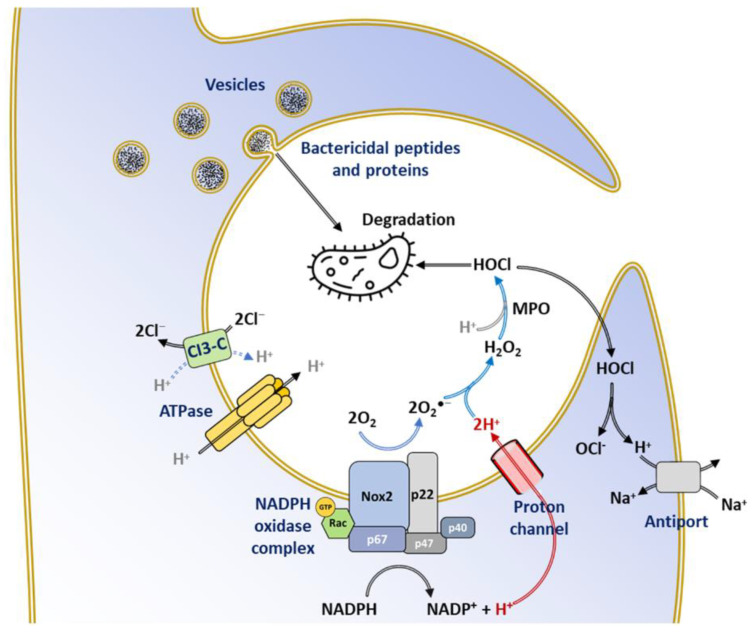
Main roles of ROS produced by NOX2 during the oxidative burst. During phagocytosis, a bacterium is sequestered in the phagosome triggering the release of bactericidal content from several types of vesicles. NOX2 transmembrane electron flow is balanced by proton flow through voltage-gated proton channels. This provides protons to the phagosome interior for the conversion of O_2_^●−^ to H_2_O_2_ and HOCl and relieves the cytoplasm of protons released by the oxidation of NADPH. Other transporters, including ClC-3 (a Cl/H^+^ antiporter), H^+^ -ATPase and the Na^+^/H^+^ antiporter, also contribute to pHi recovery. Adapted from [239,240].

Eventually, the downstream events of phagosome formation, assembly of the NOX2 complex, fusion of cytosolic granules to the phagosome, and ROS production lead to the destruction of the internalized pathogens (Figure 14).

Electron transfer catalyzed by NOX across the membrane leads to an acute membrane depolarization [236]. This phenomenon is exacerbated by the release of protons during oxidation of NADPH to NADP^+^, which also lowers the cytosolic pH [237,238]. In such conditions, the oxidative burst would then be impossible to achieve. Thus, continued NOX activity relies on the establishment of a compensatory electrogenic transfer coupled to the relief of pH at the phagosome level [237].

Proton channel activity in the transmembrane domain of NOX was initially hypothesized to explain the transfer of H^+^ compensating for the charges in neutrophils; such activity was even reported [71], although many contradicting reports suggested the existence of a separate proton channel [71,241,242,243]. The identification of a specific gene for the voltage-gated proton channel Hv1 [236,239,244,245], and the effects on NOX activity, neutrophil function, and primary immunity [246,247,248] in mouse knockouts of Hv1, put the initial hypothesis to rest. The proton channel also displays a major contribution to phagosomal pH regulation, which provides optimum phagosomal protease activity and supplies the H^+^ ions required for the conversion of O_2_^●−^ into H_2_O_2_ and HOCl [240,249] (Figure 14). Phagosomal imaging provided evidence for Hv1 as the first responder in pH regulation during phagocytosis [241] rather than the Na^+^/H^+^ antiport as believed before 1980.

#### 7.1.2. Inactivation of Virulence Factors

Besides directly contributing to the clearance of pathogens, ROS derived from NOX selectively inactivate bacterial virulence factors as an alternative to direct bacterial killing. In certain bacteria, strains such as *Staphylococcus aureus,* HOCl derived from superoxide anion generated by NOX oxidizes and inactivates quorum sensing peptides, thus promoting the virulence of microorganisms [250].

#### 7.1.3. Limitation of the Inflammatory Response

**Figure 15:**
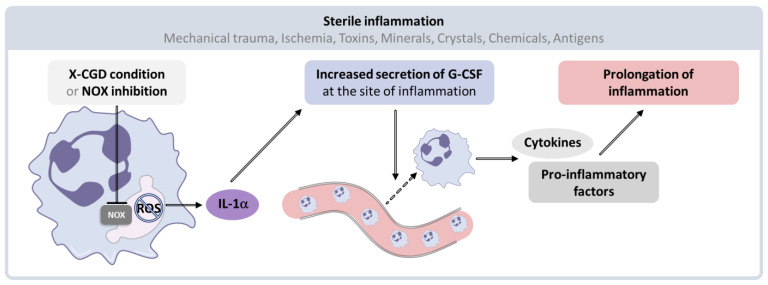
Mechanism of inflammation in the absence of NOX activation. Sterile inflammation can be triggered by physical, chemical, or metabolic noxious stimuli. In these conditions, the lack of phagocytic NOX activity leads to the overproduction of IL-1α by tissue-resident macrophages, promoting the local production of G-CSF, which induces an excessive infiltration of neutrophils and monocytes at the inflammation site. This leads to increased production of cytokines and pro-inflammatory factors that prolong the inflammation, ultimately resulting in tissue damage. Inspired by [257].

A large increase in ROS level or a decrease in the cellular antioxidant capacity can overcome the antioxidant defense system, resulting in oxidative stress. The development of oxidative stress within the cell results in a modification of cellular redox balance in favor of oxidation processes. Consequently, multiple components of the cell undergo acute ROS-mediated damage. Clear evidence underlined that the production of ROS by enzymes such as MPO or NOX yield cell and tissue injury through direct or indirect ROS-mediated damage of nucleic acids, proteins, and lipids, ultimately contributing to chronic inflammation underpinning many neurodegenerative, cardiovascular, and metabolic diseases [251,252,253,254,255]. Although the action of ROS is generally associated with pro-inflammatory activity, studies have reported an anti-inflammatory feature of NOX in sterile inflammation [256]. Notably, NOX enzymes have been shown to exert a crucial role in the interleukin-1-alpha (IL-1α)/Granulocyte Colony Stimulating Factor (G-CSF) regulatory pathway that triggers the mobilization of neutrophils in tissue lesions (Figure 15). Interestingly, a decline in NOX enzymatic activity results in early local overproduction of IL-1α in the injured tissues, then inducing the release of G-CSF by the cells at the inflammatory site. This signaling cascade thus contributes to neutrophil recruitment and ultimately to prolonged inflammatory activity [256].

#### 7.1.4. NET Activation

NETs (Neutrophil Extracellular Traps) constitute networks of extra-cellular fibers mainly composed of DNA and granular proteins produced by neutrophils to entrap micro-organisms in order to limit infectious spread [258,259]. NETs also operate a bactericidal function thanks to the joint action of proteins—such as lysozyme, proteases, defensins or histones—attached to their surface, which disrupt the membrane permeability of bacterial cells, thus leading to pathogen destruction [259,260]. This process, referred to as netosis, targets extracellular microorganisms and plays an essential role to clear pathogens which appear too large to be properly internalized by neutrophils.

Work using CGD neutrophils, p47^phox−/−^ mice, and NOX inhibitors showed convincingly that netosis can require NOX-generated ROS [261,262,263,264]. However, neutrophil extracellular trap formation in both gene-therapy restored and CGD neutrophils depended on the stimulus [265], emphasizing that different stimuli elicit different netosis response pathways, resulting in both NOX-dependent and NOX-independent netosis [263,266,267,268].

#### 7.1.5. DUOX and Other NOXs in Host Defense

While NOX2 occupies a central site among NOX enzymes in innate immunity, it is not the only NADPH oxidase isoform involved in response to pathogens [269]. The expression pattern of DUOX enzymes along the mucosal surfaces of the gastrointestinal tract and the airways suggested their participation in innate immune response. In airway mucosal surfaces, both DUOX1 and 2 serve as a source of H_2_O_2_ from which lactoperoxidase generates microbiocidal compounds via the oxidation of thiocyanate and iodide [270]. Works in Drosophila also contributed to document the role of Duox in host defense. Work in Drosophila has also helped to document the role of Duox enzymes in host defense. In flies, the silencing of Duox, a DUOX homolog, led to increased infection by gut microbes and thus to fly mortality. These effects were reversed after compensation by the reintroduction of the Duox enzyme, confirming the critical role of this enzyme in gut immunity [271].

NOX1 participates in mucosal immunity and inflammation as suggested by its expression in colon epithelium and its capacity to partially replace NOX2 [272,273]. NOX4 acts as a downstream effector of toll-like recepter 4 (TLR4), a pathogen recognition receptor. Indeed, LPS-engagement triggers an interaction between C-terminus of NOX4 and the TIR domain of TLR4 [274,275]. This functional link between NOX4 and TLR allows for the regulation of signaling pathways leading to the activation of transcription factors, as NF-kB, IRF-3, involved in the innate immune response [276].

### 7.2. Role of NOX in Redox Signaling

The discovery of NOX isoforms in multiple non-phagocytic cell types suggests a crucial impact of NOX in redox signaling [88]. Redox signaling in cells by reactive oxygen species (ROS) occurs mainly through hydrogen peroxide (H_2_O_2_) promoting S-glutathionylation and/or the reversible oxidation of cysteine thiol groups. Such oxidation can result in structural modification leading to the selective regulation of protein functions (as in phosphatase, kinases, etc.) and can thus have an important impact on regulating signaling pathways in a large range of physiological contexts [277,278,279,280].

#### 7.2.1. Regulation of Signaling Pathways

##### Inhibition of Phosphatases

The oxidation of thiols of cysteine residues constitutes the most extensively studied and probably the most important pathway in the regulation of multiple cell functions involving ROS [281], and more specifically ROS derived from NOX. This has been clearly demonstrated through the characterization of protein tyrosine phosphatases (PTPs), which control the phosphorylation state of a wide range of signal-transducing proteins, consequently regulating cell proliferation, differentiation, survival and motility [282]. Cysteines located in the vicinity of the PTP catalytic site undergo mediated oxidative inactivation [283,284], leading to a loss of phosphatase activity, jointly increasing the phosphorylation rate and thus modulating signal transduction [285]. Consistent with this inactivation process, NOX-produced ROS participates in the regulation of protein phosphorylation in different cell types [286,287].

##### Activation of Kinases

Exposure of cells to hydrogen peroxide can trigger phosphorylation and activation of MAPKs, themselves responsible for downstream phosphorylation required for cell signaling [89]. Numerous studies implicate NOX in the activation of MAPK elements [288,289,290]. On the other hand, the cascade of redox steps incriminated remains uncertain. Activation of MAPKs may result from signaling pathways upstream of ERK1/2 kinases or by the peripheral consequences arising from the ROS-mediated inhibition of phosphatase activity [89].

#### 7.2.2. Regulation of Calcium Ions

The concentration of Ca^2+^ ions represents a crucial factor supporting cellular communication. While calcium pumps maintain Ca^2+^ concentration in resting cells at an extremely low level (100 nM), a wide range of stimuli are likely to increase this concentration to micromolar levels, thus resulting in the activation of Ca^2+^ ion-dependent processes. NOX regulates at least three types of proteins involved in Ca^2+^ homeostasis (membrane calcium channels, intracellular calcium release channels, and Ca^2+^ pump), either through ROS-dependent post-translational modifications (cysteine oxidation, S-glutathionylation) or by membrane depolarization induced by electron transfer [291].

##### Membrane Calcium Channels

**Figure 16:**
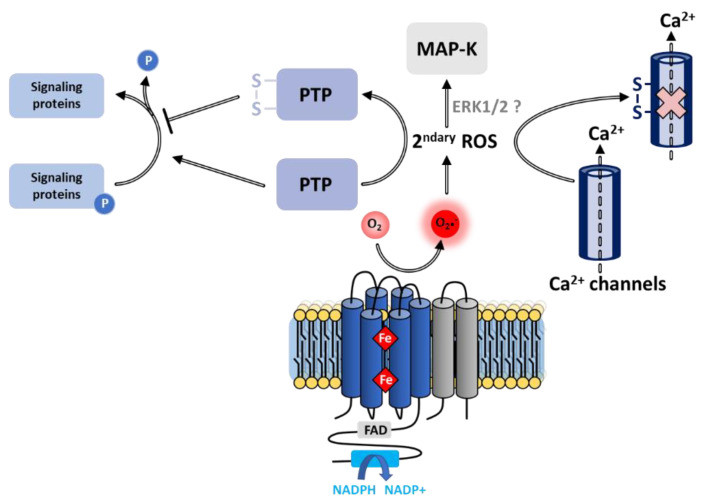
Regulation of phosphatase, kinase and calcium channel signaling pathways by NOX. The NOX-mediated production of superoxide and resulting secondary ROS leads to the oxidation of cysteines on PTPs [295] and calcium channels, resulting in the formation of disulfide bridges. PTP activity opposes that of kinases, thus regulating a large number of proteins involved in a variety of signaling pathways; PTP activity also regulates Ca^2+^ ion flux. Secondary ROS can also activate MAP-Ks, possibly via the ERK1/2 signaling pathway through activation of epidermal growth factor (EGF) receptors, and platelet-derived growth factor (PDGF) receptors, which can stimulate Ras and the subsequent activation of the ERK pathway [296].

NOX-derived ROS regulates several calcium ion transporters embedded in the plasma membrane. Oxidation of cysteine residues in the pore-forming α1-subunit of voltage-dependent Ca^2+^ channels by NOX-generated ROS modulates the opened/closed conformations of the pore, thus regulating the influx of Ca^2+^ ions [291] as well as the regulation of activity (Figure 16). Typically, ROS utterly suppresses the L-type Ca^2+^ ion flux in ventricular myocytes [292]. 

Conversely, the superoxide produced by NOX1 stimulates the Ca^2+^ flow across L-type or T-type voltage-gated channels located in smooth muscle cells. The Ca^2+^ ions involved in cell signaling originate either from an overflow from the ER, the main extracellular Ca^2+^ stores, or from a transfer through the channels of the plasma membrane. Non-selective Transient Receptor Potential (TRP) cation channels and SOC (Store-Operated Ca^2+^) represent the two main types of channels regulating the influx of incoming ions into the cell [293]. In pulmonary hypertension, NOX4 induced cell proliferation by stimulating the expression of TRPC1 and TRPC6 in response to the presence of Bone Morphogenic Protein 4 (BMP4) in pulmonary artery soft muscle cells. In addition, endothelial NOX2-derived superoxide activates TRPC6, thus triggering the Ca^2+^ influx following pulmonary ischemia reoxygenation [294].

Radical species generated by NOX also participate in modulating the SOC incoming calcium flow regulated by the translocation of the ER Ca^2+^ sensors STIM1 and STIM2 (Stromal Interaction Molecule) toward the plasma membrane. The binding of the specific sensor STIM1 to plasma membrane calcium release-activated calcium channel protein 1 (ORAI1) activates Ca^2+^ permeable ORAI1 channels, subsequently stimulating calcium entry and allowing for the regeneration of extracellular stores. The production of hydrogen peroxide by NOX2 induces the S-glutathionylation of STIM1 at cysteine56, leading to the clustering of STIM1 and activation of the SOCE (Store-Operated Calcium Entry) mechanism [294].

##### Release of Intracellular Calcium Ions

Likewise, ROS can induce the release of the Ca^2+^ stored in intracellular reserves to increase the concentration of free calcium ions [297,298]. The channels involved in this transfer belong to the ryanodine receptors family (RyRs) that possess redox-sensitive cysteine residues [299]. Activation of Ca^2+^ channels has been demonstrated not only by the exogenous addition of H_2_O_2_ [300,301], and superoxide anions [302], but also in response to NOX-dependent ROS production [303,304,305]. However, it would seem that NOX-derived ROS does not systematically induce global cellular Ca^2+^ increases but instead triggers a targeted mode of action referred to as Ca^2+^ sparks [303], leading to rapid transient Ca^2+^ inflows with restricted localization. RyRs organized into clusters of variable size from a few to several hundred RyRs, creating a spatially nonuniform intracellular distribution; however, it is unclear how the heterogeneity of RyR cluster size alters spontaneous Ca^2+^ sparks [306]. ROS are also implicated in the activation of intracellular Ca^2+^ channels of the IP3 receptor family [307].

##### Calcium Pumps

Finally, the activity of Ca^2+^ ATPase pumps can also be modulated by the concentration of ROS according to two distinct mechanisms [297,308,309]:

- At low concentrations of ROS, the *S*-glutathionylation of cysteine residues by interaction of glutathione with peroxynitrite radicals leads to the formation of reversible disulfide bridges that stimulate Ca^2+^ pumps.
- At a higher ROS concentration, the excessive level of oxidative stress ends in irreversible thiol oxidation, resulting in the inactivation of enzymes [297].

#### 7.2.3. Regulation of Cell Growth and Death

##### Cell Death

A large number of studies describe cell death as a consequence of NOX activation. ROS induce apoptosis indirectly through damage to DNA, proteins and lipids, or more directly through the activation of pro-apoptotic signaling cascades such as SAPK/JNK, ERK1/2, and p38 upon the induction of the MAPK pathways [310]. At high concentrations, ROS—especially as H_2_O_2_—can inhibit caspases, thus irremediably damaging cell components and ending in necrosis [311,312].

Conversely, in certain cases, NOX-produced ROS can trigger an anti-apoptotic effect by activating NF-κb [313] or Akt/ASK1 [314] transduction pathways. The superoxide anion may be a natural inhibitor of the ligand/receptor FasL/FasR promoting cell death [315].

The distinction between pro- and anti-apoptotic functions is modulated by various factors, including: the intensity and duration of the redox signal; the cellular localization of the NADPH oxidase responsible for the ROS production; the type of ROS generated; and the targets of redox signals expressed by the cell.

##### Cell Growth

In contrast to the role in cell senescence, ROS also share in the regulation of cell growth. The insight that developing tumor cells produce excess ROS [74] initiated various studies describing ROS as potential secondary messengers and established their role in the stimulation of cell proliferation [310,316,317,318]. Earlier studies noted the lack of proliferation defects in NOX2-deficient patients, casting doubt, but the discovery of the other NOX homologs clarified the issue. NOX1-derived H_2_O_2_ involvement in cell proliferation was finally established both in situ and in vitro [46,319]. In vitro assays based on antisense or small interfering RNAs suggested that NOX5 stimulates the proliferation of smooth muscle cells and NOX4/1 stimulates the proliferation of esophageal cells [320]. Similarly, the inactivation of p22^phox^ and NOX2 RNAs underlined that these proteins play a key role in the regulation of cell growth [321]. Finally, recent work showed the role of NOX4 in hematopoietic differentiation [322].

#### 7.2.4. Role in Biosynthesis Mechanisms

The iodination step of thyroid hormone biosynthesis constitutes a well-documented role of DUOXs belonging to the NOX family. Thyroid-localized DUOX generates H_2_O_2_, which thyroid peroxidase uses as a source of oxidant for the iodination of tyrosine residues of thyroglobulin, leading to the synthesis of T3 and T4 hormones [47,48]. A recent review is available for update information on the role of DUOX and H2O2 generation in thyroid cells [323]. Mutations in the DUOX2 gene can lead to a form of congenital hypothyroidism resulting from the absence of H_2_O_2_ and a consequent inability to produce thyroid hormones [324].

NOX3 participates in a different biosynthetic process in the inner ear of vertebrates. Here, superoxide produced by NOX3 activates the morphogenesis of otoliths, mineral concretions that ensure the perception of gravity and the maintenance of balance [325]. As evoked in a section above, in addition to its role in balance, NOX3 is involved in hearing loss and is believe to be a good target for inner ear pathologies [326,327].

#### 7.2.5. Role in Angiogenesis

**Figure 17:**
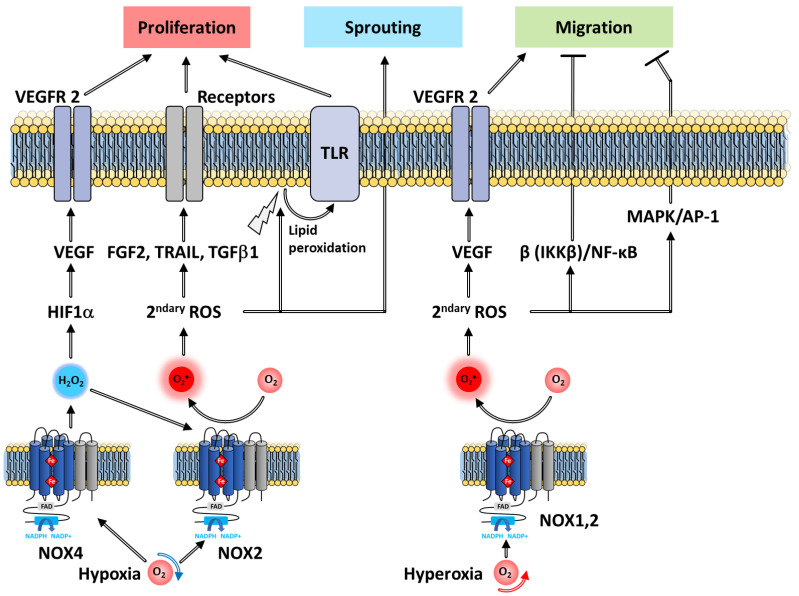
Regulation of angiogenesis steps by NOX. Hypoxia conditions activate NOX4 and NOX2, inducing the production of ROS and thereby enhancing VEGFR2 signaling and angiogenesis in ECs. Nox4-derived H_2_O_2_ also activates NOX2 to promote superoxide production. NOX-mediated ROS promotes lipid peroxidation activating TLR, which regulates cell proliferation. Hyperoxia conditions activate NOX1, leading to the elicitation of cell migration through the VEGF pathway. The ROS-mediated β(IKKβ)/NF-κβ and MAPK pathways can inhibit this process.

Angiogenesis is a finely tuned process in which new capillaries develop from pre-existing blood vessels. Both pro- and anti-angiogenic factors modulate this process by acting on endothelial cells (ECs). Resting ECs respond to pro-angiogenic factors that stimulate the expression of growth factor receptors as well as the release of proteases promoting membrane destabilization. Subsequently, ECs proliferate and sprout, migrating towards the angiogenic stimulus as they grow into tubules with a lumen. Multiple angiogenesis signals activate NOX 1,2,4 and 5, which participate in the angiogenic response of the ECs (Figure 17).

##### The Proliferation Stage

Since 1999, NOX1 has been known to stimulate the proliferation of various cells [46], including vascular smooth muscle cells (VSMCs). A recent study revealed that NOX1 inactivation impairs the hypoxia-induced proliferation of ECs from pulmonary arteries [328], demonstrating an important role of NOX1 in this process.

NOX2 and NOX4 overexpression induces ROS production and EC proliferation, whereas their inactivation inhibits both of these processes, thus demonstrating the direct relationship of NOX2 and NOX4 activity to these phenomena [329]. Induction of NOX4, promoted by hypoxia conditions as well as various growth factors, such as vascular endothelial growth factor (VEGF) [330], basic fibroblast growth factor (FGF-2), TNF-related apoptosis-inducing ligand (TRAIL) [331], or transforming-growth-factor-β1 (TGFβ1) [332,333], can enhance the growth of human microvascular ECs. Furthermore, it has been suggested that NOX4-derived hydrogen peroxide induces subsequent activation of NOX2, ultimately resulting in the activation of VEGF receptors [329,333,334,335] and stimulating EC proliferation. These results testify to the existence of a proper coordination between the NOX isoforms necessary for angiogenesis.

##### The Sprouting Stage

Various investigations of tumor angiogenesis demonstrated the involvement of NOX in EC sprouting. Thus, the aortas of transgenic mice overexpressing NOX4 (NOX4 TG) exhibit a 25% higher rate of EC sprouting than the ECs of wild-type aortas [336]. Angiopoeitin-1 (Ang1), an important regulator in vascular angiogenesis, stimulates the sprouting of ECs. Conversely, the p47^phox−/−^ CGD rodent model, disabling the expression of the NOX cytosolic subunit, downregulates this process, as does a wide range of NOX inhibitors, highlighting the involvement of NOX in the angiotensin-mediated regulatory pathway of Ang1.

Sprouting was also observed in models of pig coronary artery endothelial spheroids exposed to hypoxia/re-oxygenation [337]. However, these effects were decreased in p47^phox^ knockout spheroids, implicating NOX cytosolic proteins in sprouting [337]. In a second model, aortas of p47^phox−/−^ mice had impaired sprouting ability compared to the wild type in response to hypoxia and re-oxygenation [337].

Furthermore, despite stimulation by urotensin II, EC budding in NOX2^−/−^ mice failed to be restored. Taken together, these studies demonstrated that NOX2, NOX4 and p47^phox^ are involved in the sprouting of ECs.

##### The Migration and Tubule Formation Stages

The role of NOX1 in the regulation of tubule formation has been extensively described using ECs from the lungs of NOX1-deficient mice that are unable to produce ROS after stimulation by VEGF and FGF-2 growth factors [338]. Under these conditions, ECs are unable to migrate to form tubules. On the other hand, the expression of Peroxisome Proliferator-Activated Receptor α (PPARα) was observed in the deficient cells and further experiments have shown that exposure of these cells to GW6472, a PPAR antagonist, successfully restored angiogenesis capacities [338].

The migration of ECs is often initiated upon detection of a hypoxic environment [339] but can also be initiated in an environment of excess oxygen. Pendyala et al. (2009) found that the hyperoxia-stimulated production of ROS and EC migration was, in part, due to NOX2 in human lung microvascular ECs [340]. Selective inhibition of NOX2, either mediated by an adenovirus or by using p47^phox^*^−/−^* mouse models, altered the stimulation of Ang1-mediated EC migration [338]. Moreover, the impairment of Ang1-induced EC migration was also observed in ECs isolated from p47^phox^*^−/−^* mice, thus confirming the involvement of NOX in this process [340].

On the other hand, through IκB kinase-β(IKKβ)/NF-κβ and MAPK/AP-1 (Activator Protein 1) regulatory pathways, NOX2 contributes to the attenuation of LPS stimulated endothelial cell tubule formation [341]. 

Stromal cell-derived factor-1 (SDF-1α) is a potent angiogenic chemokine inducing the migration of human microvascular EC [342]. Silencing p22^phox^ or NOX-5 by siRNA inhibited the migration induced by SDF-1α [343] and also significantly reduced SDF-1α-induced tubule formation after 72 h. This suggests that NOX5 and NOX isoforms involving the p22^phox^ subunit are involved in SDF-1α migration [343].

## 8. Pathologies Related to NOX Deregulation

The ROS produced by the NOX family participate in a variety of physiological mechanisms beyond immune defense, most significantly a large number of signaling pathways that regulate crucial biological functions such as angiogenesis, cell proliferation and apoptosis. The loss of ROS homeostasis directly affects these processes, leading to human pathologies [344].

### 8.1. Chronic Granulomatous Disease

As described in Section 2, CGD pathology is the result of an inactive NADPH oxidase complex leading to a loss of bactericidal activity of neutrophil, an inability to fight again infections and thus to an inherited innate immunity deficiency. The most common and severe form of CGD (CGD-X) arises from mutations in the NOX2-encoding gene *CYBB* on the X chromosome. Analysis of X-CGD-related mutations revealed the existence of three distinct cases defined by a total absence of NOX2 (X^0^-CGD), or by a low expression of the mutated protein correlated with a reduced oxidase activity (X minus-CGD), or finally by normal expression of NOX2 but a loss of oxidase activity (X+CGD) [345]. These alternatives provided crucial models that led to definitions of functional domains and residues in NOX2 [346]. X^+^ CGD-related mutations mainly affect the catalytic activity of the oxidase, while the mutations responsible for X minus CGD appear to affect the proper maturation and correct folding of NOX2 [347].

The most widespread form of CGD (X^0^-CGD) has been characterized by an absence of the two subunits of cytochrome b_558_, although only the *CYBB* gene expression was demonstrated to be affected with reduced or undetectable levels of mRNA [348]. This close interdependence between NOX2 and p22^phox^ subunits has been confirmed in Autosomal Recessive (AR) CGD, where a p22^phox^ deficiency resulted in the absence of NOX2 [94,349] as well as in specific X minus CGD mutations [350,351].

Mutations in genes for other proteins in the NOX2 complex (p47^phox^, p67^phox^, and p22^phox^) result in AR forms of CGD [352]. Both X-linked (X^0^-CGD) and autosomal (AR^0^-CGD) CGD mutations generally cause the absence of the associated protein, stemming either from defective synthesis of the messenger RNAs or reduced protein stability, leading to rapid elimination [353].

### 8.2. Central Nervous System Diseases

The brain includes a variety of oxidation-sensitive lipids but also has reduced antioxidative defense mechanisms. This, combined with the large amount of oxygen consumed by the brain, confers to this organ an acute sensitivity to the misregulation of redox homeostasis [354]. As a consequence, alterations in the ROS production pathways can result in a wide range of neurological disorders and significant damage to this organ. NOX1, 2, 3 and 4 are expressed in cells of the central nervous system (CNS) such as neurons [355], microglia [356,357] and intracranial vessels [358]. Many studies have documented the relationship of NOX enzymes with degenerative diseases of the brain [354,355].

#### 8.2.1. Parkinson’s Disease

Parkinson’s disease (PD) is a neurodegenerative disorder characterized by a stepwise destruction of the dopaminergic neurons in the nigrostriatal pathway of the brain; this destruction triggers complex functional modifications within the basal ganglia circuitry, ultimately leading to motor dysfunctions [359].

Research on PD mainly relies on the development of a mouse model in which the administration of 1-methyl-4-phenyl-1,2,3,6-tetrahydropyridine (MPTP) produces the PD-like symptoms of human degeneration [360,361]. MPTP signals the translocation of p47^phox^ to the membrane, and subsequently the activation of NOX2 [362]. Increased levels of p47^phox^-NOX2 complexes were detected in vivo after systemic injections of MPTP [363]. Additionally, it was notably demonstrated that the CNS, particularly dopaminergic (DA) neurons, is prone to oxidative damage, resulting in cell degeneration and PD pathogenesis [364,365].

In agreement with these findings, NOX2^−/−^ mice given MPTP showed attenuated damage to DA neurons compared to WT counterparts, supporting NOX2’s function in the PD-related loss of dopaminergic neurons [359].

**Figure 18:**
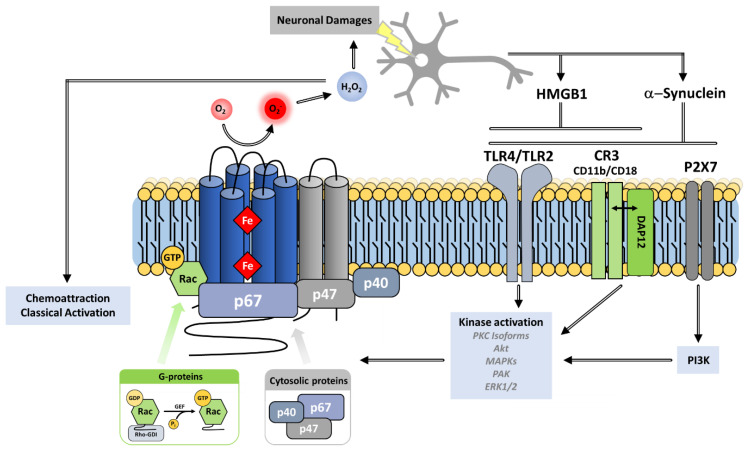
Activation mechanism of microglial NOX2 involved in Parkinson’s disease. a-Synuclein-induced micro-glia activation may involve different surface receptors such as P2X7, TLR2/4 and CR3 that activate kinases, leading to phosphorylation of p47^phox^ and NOX2 activation. Subsequent ROS production leads to migroglial chemoattraction and oxidative stress. Resulting neuronal damage then further activates the release of the protein (HMGB1) and α−synuclein. Figure is adapted from [366].

A microglial expression of the NOX enzymes involved in the PD disease is evidenced by immunostaining assays (Figure 18). 

Additional data suggested that NOX are also involved in neurodegenerative symptoms induced by 6-hydroxydopamine (6-OHDA), yielding an acute production of superoxide anion correlated with a joint enhanced expression of NOX2 and p47^phox^ [367].

Finally, the involvement of NOX in PD has also been revealed through other unrelated models. Thus, it has been reported that the NOX inhibitor diphenyleneiodonium (DPI) blocked paraquat-induced ROS production and subsequent DA neurodegeneration [368].

#### 8.2.2. Alzheimer’s Disease

Alzheimer’s disease (AD) arises from a stepwise neuronal decline that originates in the hippocampus, a cerebral structure essential for memory. As this decline extends in the brain, it leads to a dramatic loss of higher cognitive functions resulting in dementia. Accumulation of amyloid-β peptide (Aβ) in the brain is generally considered one of the main pathological indicators of AD [369].

Several lines of evidence indicate that NOX contributes to this pathology. In a rodent model, exposing microglial cells to high concentrations of Aβ peptides induced the translocation of NOX regulatory subunits, while inhibiting NOX2 activity with gp91ds-tat peptides diminished degenerative symptoms [370]. The oxidation of cholesterol into 24-hydroxycholesterol, promoted by redox imbalance and more specifically by the large amount of H_2_O_2_, potentiates both the pro-apoptotic and pro-necrotic effects of Aβ [371].

**Figure 19:**
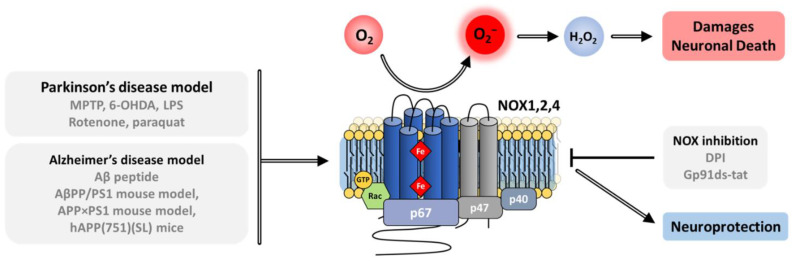
NOX-mediated neurodegeneration and neuroprotection. In Parkinson’s and Alzheimer’s disease models, the ROS overproduction resulting from the activity of NOX family contributes to neurodegeneration. NOX inhibition mediated by apocynin, DPI or Gp91ds-tat induces neuronal protection. Figure is inspired by [376].

The brains of AβPP/PS1 double transgenic mice, a mouse model of AD, showed a significantly higher expression of NOX2 and NOX4 [372]. Treating these mice with phenolic antioxidant tert-butylhydroquinone inhibits NOX2 expression and thereby prevents the cerebral cortex and hippocampus from lipid peroxidation [373], revealing the existence of a significant linear relationship between NOX activity, Aβ production rate, and neuronal decay. In the bilateral cerebral artery occlusion (BBCAO) rodent model of early-stage vascular dementia, upregulated expression of NOX1 and NOX3 mRNAs and corresponding high levels of superoxide have been described [374] in the hippocampus CA1 region responsible for neuronal dementia [375]. Taken together, these findings indicate that NOX enzymes play a role in the development of AD (Figure 19).

### 8.3. Cancers

Several NOX, and their regulatory subunits, show markedly increased expression in many types of human tumors or cancer cell lines cultured at different stages of tumorigenesis, suggesting NOX participation in these events [377,378,379]. Similarly, several studies over a large number of patients suffering from gastric cancers (GC) also showed that high levels of NOX2/4, and DUOX1 at the tumor site, compared to adjacent tissues, constitute reliable prognostic markers in GC [380,381].

Athymic mice with exogenous expression of NOX1 in wild-type fibroblasts of the NIH3T3 cell line presented noticeably enhanced cell growth and tumor formation [46]. In these experiments, NOX1-transfected cells (10-fold over-expression of NOX-1 in NIH3T3 fibroblasts) induced increased growth and transformation despite a restricted production of superoxide anions, revealing that high levels of ROS are not responsible for the initiation of tumor processes [319]. However, NOX1 produces a marked increase in intracellular H_2_O_2_, formed from the dismutation of O_2_ and the coexpression of catalase (CAT) promoting the recovery of the initial phenotype. This demonstrated that H_2_O_2_ is nonetheless a likely accountable factor for the induction of these mechanisms [319,379].

**Figure 20:**
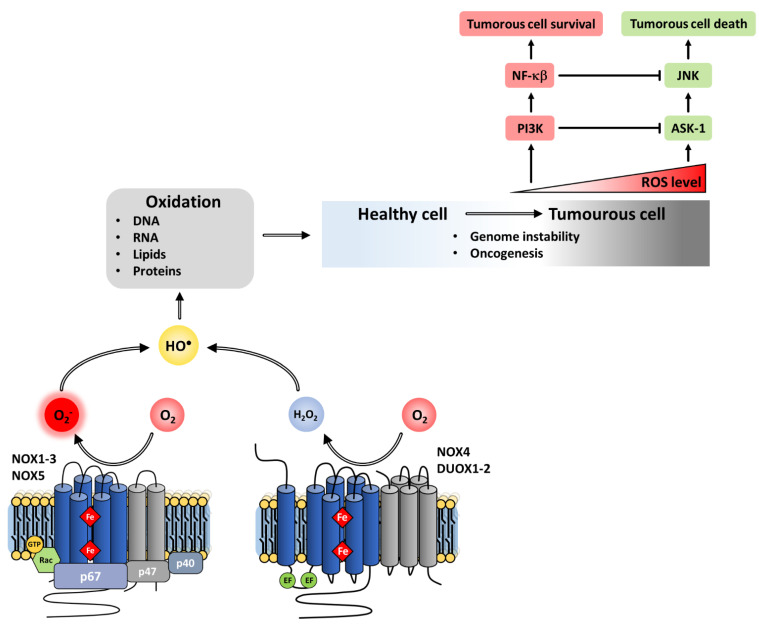
Endogenous sources of ROS and overproduction-related consequences. The overproduction of superoxide anions and H2O2 by NOX enzymes and the subsequent increase in hydroxyl radical levels through Fenton and Haber–Weiss reactions lead to oxidation of lipids, proteins and DNA, and consequently promotes genomic instability, high mutation rate and carcinogenesis. Under these conditions, cell survival or cell death, respectively, depend on the activation of the PI3K or Ask-1 signaling pathways. High levels of ROS stimulate the Ask-1/JNK pathway, leading to cell death, while lower or transient levels of ROS may activate PI3K kinases and Ask-1/JNK inhibition, thus ensuring NF-κβ-mediated cell survival [385]. Inspired by [385].

A putative role of NOX-derived hydrogen peroxide as anti-tumor agent has also been suggested following the description of an H_2_O_2_-dependent activation of apoptotic cell death after treatment with doxorubicin or camptothecin [382,383,384] (Figure 20).

#### 8.3.1. Tumor Development

The effect of ROS in DNA damage has been extensively reviewed; cell exposure to chronic oxidative stress has been reported to elicit genomic instability [386,387], and there is evidence for increased levels of ROS [388,389] in genomically unstable clones. In this context, NOX-derived ROS is a logical contributor to this phenomenon. Although the exact role of NOX in cellular transformation remains unclear, several studies furnish suggestive evidence.

H_2_O_2_ produced by NOX4 damages mitochondrial DNA and induces mitochondrial dysfunction [83,390,391]. NOX4 was also presumed to be responsible for the direct oxidation of nuclear proteins and DNA as indicated by NOX4 localization within the nucleus [392].

Along with the generally attributed role of NOXes in chromosomal instability, NOX 1, 2, 4 and DUOXes have been linked to the regulation of p53 transcription factor activity. Attributed since 1989 to tumor suppression, the gene associated with p53 cell cycle inhibitor appeared to be inactivated in 50% of the human cancers. Several kinds of evidence make clear an extensive crosstalk between NOX4 and p53, in which each affects both the expression and activity of the other, ultimately influencing tumor formation and progression [393,394,395]. The homeodomain-interacting protein kinase 2 (HIPK2) corepressor upregulates NOX1, inhibiting Sirtuin1 (SIRT1) and thus indirectly inhibiting the deacetylation and inactivation of p53 [396]. The induction of NOX1 expression is also connected to an increase in mutation rate in the proto-oncogen K-RAS, involved in 30% of human tumors [397].

#### 8.3.2. Proliferation, Invasion and Metastasis

**Figure 21:**
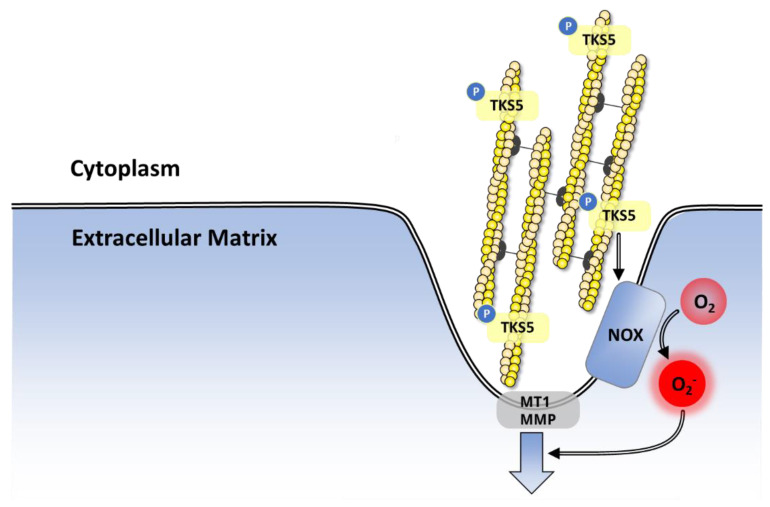
Invadopodia regulation model modulated by the association of Tks5 with NOX. Initial model proposed by [406] was further adapted to include the formation of Tks-5-NOX complex assembly and subsequent activation of ROS-mediated invapodia formation. Inspired by [406].

Cancerous cells spread and proliferate via a sequential metastatic cascade featuring the invasion of the extracellular matrix by tumor cells, followed by a stepwise migration through the endothelium towards vessels (intravasation/extravasation), colonization, and initiation of a secondary tumor [398,399]. Invadopodia, actin-rich structures mainly containing integrins and metalloproteases, mediate extracellular matrix degradation and extravasation steps. Invadopodia formation relies on superoxide produced by NOX [400,401,402]. Proteins Tks4 and Tks5, which have some similarity to p47^phox^ and are exclusive to invadopodia, bind and activate NOX1 and NOX3 independent of the usual NOX cytosolic subunits [403,404]. Proper assembly of the NOX-Tks protein complex exclusively found in the membranes of these structures appears essential for invadopodia formation [400,405] (Figure 21).

Evidence exists for TLR stimulation of NOX1 in cancer cells. In lung cancer cells, TLR signaling stimulated NOX1-derived superoxide that regulates metastasis [407]. It is supposed that NOX1 could mediate the expression of C-X-C chemokine receptor type 4 (CXCR4) and Matrix metallopeptidase 9 (MMP9) [407,408], which play an important role in the metastasis of non-small-cell lung carcinoma (NSCLC) [408,409]. In colon, NOX1 may accelerate the adhesion of LPS-stimulated cancer cells through a mechanism in which TLR-4-mediated activation of NF-κβ leads to increased activation of NOX and a higher level of ROS, promoting the phosphorylation of Akt [410]. In bladder cancers, the PI3K/Akt signaling pathway mediates TG-interacting factor- (TGIF-) induced NOX2 activation and superoxide production, which stimulate PI3K/Akt to promote the invasiveness of urothelial carcinoma [411]. 

#### 8.3.3. Tumor-Mediated Angiogenesis

**Figure 22:**
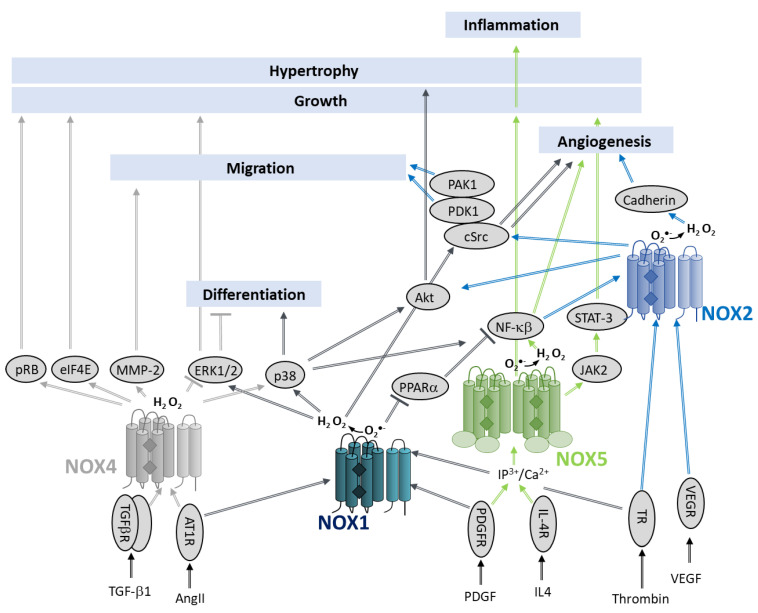
Examples of signaling pathways involving NOX1, 2, 4 and 5 in the regulation of the different tumorous stages. NOX1-derived superoxide, activated by thrombin, PDGF or Ang II, can affect cell differentiation via the p38 and ERK1/2 pathways, hypertrophy via p38-mediated Akt activation, cell migration via cSrc (Proto-oncogene tyrosine-protein kinase Src) activation and cell growth via ERK1/2 activation, subsequent activation of transcription factor Ets-1 and upregulation of cyclin D. NOX1 also downregulates the expression and activity of the antiangiogenic receptor PPARα, which is known to inhibit the NF-κB transcription factor and thus angiogenesis. NOX2, activated by TNFα, thrombin or NF-κB-induced ROS, promotes migration and angiogenesis via the Akt and cSrc pathways. NOX2-mediated regulation of angiogenesis also occurs via cadherin, p38 activation or regulation of ERK1/2 activation mediated by NOX4-derived H_2_O_2_. NOX2 also regulates cell growth via p38 activation or inhibition of ERK1/2 differentiation, and migration via the eiF4E (Eukaryotic translation initiation factor 4E) and pRB pathway. NOX4 expression and activity are promoted by TGF-β1 or Ang II. NOX5, whose superoxide production is activated by IL4 or PDGF, promotes cell growth and inflammation, respectively, through the JAK-2 / STAT3 and NF-κB signaling pathways. Inspired by [385].

The ability of cancer cells to spread to adjacent or distant tissues depends heavily on oxygen and nutrients delivered by the vascular system [412]. Angiogenesis constitutes an essential process in the development of solid tumors by ensuring the direct delivery of nutrients to cancer cell clusters. The HIF-1α (Hypoxia Inducible Factor 1α)/VEGF/MMP signaling cascade activated by hypoxia and by ROS [413,414] regulates the formation of new blood vessels promoting tumor formation. The underlying mechanisms of this process has been deciphered though the study of the degradation of HIF-1α in normal oxygen conditions, awarded the 2019 Nobel Prize in Medicine. NOX1, 2, 4 and 5 (Figure 22), localized in endothelial cells participate in every stage of angiogenesis (Part 6.2.5), and play a crucial role in cancer-induced blood vessel formation [338,413,415,416,417].

**Figure 23:**
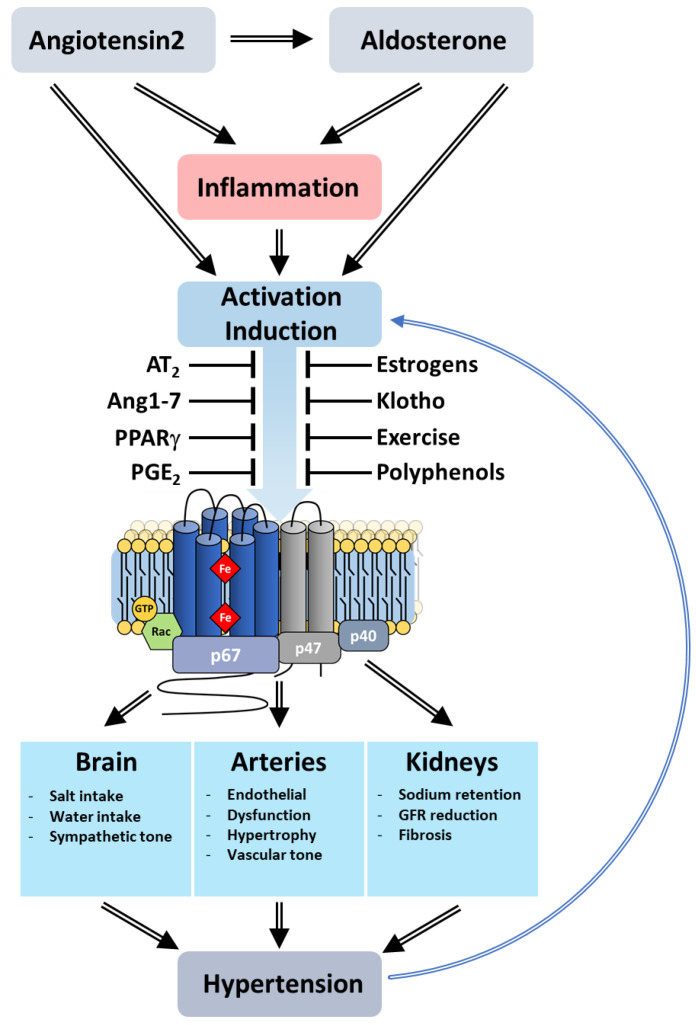
Role of NOX in hypertension. Angiotensin II leads to the activation and expression of NOX via the AT_2_ receptor, aldosterone, inflammation and increased vascular tone. Multiple factors including prostaglandin E_2_ (PGE_2_), exercise, estrogen, and angiotensin, via the AT_2_ receptor, contribute to limit this process. NOX located in the brain, arteries and kidneys promote the development of hypertension through various mechanisms. In the kidney, for instance, activation of NOX presumably destabilizes the tubulo-glomerular feedback loop and the glomerular filtration rate (GFR) [428].

In ovarian cancers, NOX4-derived H_2_O_2_ regulates HIF-1α expression which in turn governs VEGF levels, essential for tumor-induced angiogenesis [415,418]. ROS production by NOX1 and NOX4 also stimulates HIF-1α-mediated vascularization in prostate cancer and malignant melanoma [416,418]. However, it is noteworthy to mention several studies reporting that ROS-mediated angiogenesis likely occurs in an HIF-1α independent mechanism [416,418,419]. NOX1 was reported to have a role in endothelial cell migration [338] through the downregulation of the expression and activity of the antiangiogenic receptor PPAR*α* (peroxisome proliferator-activated receptor *α*), which is known to inhibit the transcription factor NF-*κ*β (Figure 23) and VEGF [338,420]. Another mechanism has been reported for serotonin-induced angiogenesis: serotonin (5-HT, 5 -hydroxytryptamine) activates NOX and induces ROS production, which is probably mediated through the activation of the 5-HT1 receptor-linked Src/PI3K pathway [421].

### 8.4. Cardiovascular Pathologies

The production of ROS in the blood vessels is essential to redirect the blood stream to the most active tissues and thus maintain vascular homeostasis. However, ROS also contributes to the development of cardiovascular diseases such as hypertension, atherosclerosis, diabetes, hypertrophy and cardiac arrest. Multiple ROS-producing enzymes—including NOX, nitric oxide synthases (NOS), respiratory complex proteins and cytochromes P450—that are unevenly distributed and expressed throughout the vascular system produce ROS. While all of these enzymes participate in various pathological conditions, NOX appears to exert a key role in modulating the stimulation or dysfunction of downstream enzymes [422,423].

The NOX expression profile in vascular cells and tissue varies depending on the specific pathology and also the stages of any disease’s progression [422]. In physiological conditions, vascular NOX present a low basal activity [424]. However, misregulation or chronic production of large amounts of NOX-derived ROS, stimulated by signals such as cytokines [425], growth factors [426] or high glucose levels [427], interferes with vascular homeostasis and promotes the development of cardiovascular pathologies.

#### 8.4.1. Hypertension

Among the first pathologies undoubtedly attributed to NOX activity (Figure 23) [429], hypertension constitutes a multifactorial pathology involving enhanced vascular resistance, increased cardiac output, decline of renal sodium excretion and dysfunction in blood pressure regulation.

Angiotensin-2 (Ang-2), which exerts a crucial role in the development of hypertension, represents a major positive regulator of the NOX-mediated production of ROS in the vascular system. Acting through angiotensin type 1 (AT1) receptors [430], Ang-2 stimulates the expression of NOX1, NOX2 and NOX4 homologues and the cytosolic factor p22^phox^, all implicated in hypertension and associated vascular dysfunction [429].

The aortas of aged spontaneously hypertensive rats (SHRs) displayed an Ang2-induced overexpression of NOX1, enhanced NOX activity, a significant increase in systolic blood pressure, and hypertrophy. The deletion of NOX1 protected SHRs from vascular dysfunction and complications [431]. Similarly, overexpression of NOX2 genes in SHRs models led to a hypertensive phenotype [422], while p47^phox^ knockout mice with low NOX2 activity exhibited diminished hypertension and preserved endothelial functions after chronic exposure to Ang2 [432,433].

While the molecular studies of NOX1 and NOX2 showed their role in promoting hypertension, NOX4 activity has been linked to a protective function [434]. For example, the overexpression of NOX4 and enhanced H_2_O_2_ production stimulated vasodilation and resulted in a reduced basal blood pressure, suggesting a protective role for NOX4 [435]. Under Ang2-induced stress conditions, Nox4^−/−^ mice exhibited impaired expression of the heme oxygenase-1 (HO-1) and endothelial NOS (eNOS). This resulted in a lower production of nitric oxide, consequently promoting apoptotic and inflammatory responses [434]. In contrast to NOX1 and NOX2, NOX4 promotes the protection of the vascular system during ischemic or hypertensive stress.

#### 8.4.2. Atherosclerosis

Atherosclerosis is a condition characterized by the accumulation of arterial plaques, mainly composed of lipids, on the walls of arteries, ultimately resulting in damage to the arterial wall and obstruction of vessels.

Animal models show that vascular cells adjacent to atheromatous plaques presented higher levels of NOX2, NOX4 and NOX5 expression than healthy cells [436,437], suggesting NOX participation.

**Figure 24:**
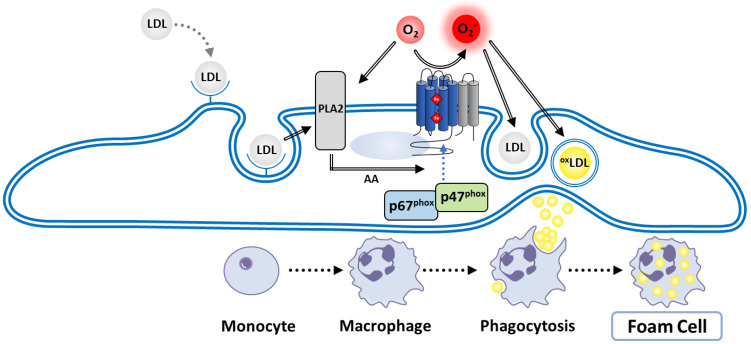
Model of the crucial role of NOX2 in atherogenic EC generation. Atherogenic LDL levels activate the re-lease of intracellular ascorbic acid via phospholipase A2, leading to the translocation of p47^phox^ and p67^phox^ to NOX2. The subsequent production of O_2_^●^^−^ by NOX leads to increased LDL transfer across the EC, during which the exposure of LDL to high levels of O_2_^●^^−^ may cause oxidation of LDL (ox-LDL) on the abluminal side of the EC. The ox-LDL thus generated is absorbed by macrophages that are attracted by the accumulation of cell adhesion molecules, thus explaining the pro-duction of foamy cells observed in the early stages of atherosclerosis [438].

Molecular-level explanations ensued. In the presence of low-density lipids (LDL), the increased binding and uptake by cell surface LDL receptors may be responsible for the direct activation of NOX. NOX-generated superoxide stimulates lipid endocytosis, thus promoting plaque formation (Figure 24).

In addition, O_2_^−^ causes an increase in the trafficking across the EC layer. LDL invaginated into endocytotic vesicles comes into contact with superoxide, generated by the EC NOX, resulting in the production of ox-LDL on the luminal side of the EC [438]. Macrophages attracted by the increased number of cell adhesion molecules then internalize oxidized lipids, accounting for the production of foam cells typically observed during the early development of atherosclerosis. The subendothelial accumulation of foam cells is also an important indicator of atherosclerosis.

The increased levels of NOX-mediated O_2_^−^ also promotes both the translocation of NF-κβ transcription factor to the nucleus and the up-regulation of a large variety of genes such as ICAM and ELAM-1 associated with the early stages of atherosclerosis [438].

#### 8.4.3. Diabetes

Diabetes is associated with a wide range of metabolic degenerations such as insulin resistance and hyperglycemia, but the majority of deaths in diabetic conditions result from cardiovascular complications.

Both animal models [439] and human diabetic patients [440] showed an increased ROS production during hyperglycemia that promotes endothelial dysfunction, further stimulating the detrimental progression of diabetes-related vascular pathologies.

NOX2 and NOX4 have been detected in the aorta of ApoE^−/−^ atherosclerotic mice models exposed to the streptozotocin diabetes inducer [441]. Likewise, NOX1 and NOX4 were found to be over-expressed in db/db diabetic mice, indicating that the NOX1 and NOX4 isoforms may exert a potential role in diabetes-related macrovascular disease [442]. Deletion of NOX1 in diabetic mice, or exposure of these animals to GKT137831 to inhibit NOX1, induced a systematic attenuation of atherosclerotic plaque formation [443]. (The reader is cautioned that this inhibitor probably does not directly inhibit NOX1 [444].) However, the absence of NOX4 did not induce significant changes in vascular conditions in diabetic mice, suggesting that this isoform does not support a direct role in diabetic vasculopathies. It may, instead, exert indirect effects through the regulation of adipogenesis [445] via the ERK1/2 MAPK signaling pathway. NOX4 gene suppression in pre-adipocytes notably blocks the differentiation of stem cells into adipocytes while high levels of NOX4 have been reported in pre-adipocytes. By switching the balance toward differentiation of preadipocytes, NOX4 promotes obesity and inherent heart disease [446]. 

Hyperglycemia also induces elicitation of vascular NOX. Indeed, contrary to wild-type cells, incubation of endothelial cells with red blood cells from patients suffering from type 1 diabetes led to activation of endothelial NOX [447]. This activation leads to enhanced amounts of ROS, as indicated by the increased production of superoxide detected in the arteries and veins of diabetic patients.

## 9. Conclusions

NADPH oxidases constitute a key target for biological science research. The present article reviews recent progress in this field and on the physiological function of the seven human isoforms of NOX enzymes, specifically in cell signaling and cell differentiation through NOX-mediated ROS secondary messengers. NOX appears to be beneficial to a wide range of physiological processes, including in innate immunity, bone remodeling, signal transduction and the biosynthesis of biologically important substances such as thyroid hormone and otoliths. Given NOX’s roles in maintaining normal physiology, it is not surprising that deregulation of these enzymes can likely induce multiple pathologies, as illustrated by extensive studies reporting various degenerative diseases of the central nervous system, cardiovascular diseases, cancer, diabetes and others. NOX family members, therefore, constitute strategic therapeutic targets. More information is required to propose efficient and reliable therapeutic solutions to remedy complications stemming from these pathologies. The development of suitable molecules as a treatment of these conditions primarily requires a thorough understanding of the regulatory processes of NOX activation mechanisms through the functional and structural study of these enzymes. The newest structural NOX information promises significant advances in this area in the years to come. 

## Figures and Tables

**Table 1:** Main features shared among the NADPH oxidase family of enzymes.

NADPH Oxidase
O_2_ consumption
Cyano-resistant
Dependent on FAD and NADPH
Presence of heme groups
